# FANCI Regulates Recruitment of the FA Core Complex at Sites of DNA Damage Independently of FANCD2

**DOI:** 10.1371/journal.pgen.1005563

**Published:** 2015-10-02

**Authors:** Maria Castella, Celine Jacquemont, Elizabeth L. Thompson, Jung Eun Yeo, Ronald S. Cheung, Jen-Wei Huang, Alexandra Sobeck, Eric A. Hendrickson, Toshiyasu Taniguchi

**Affiliations:** 1 Howard Hughes Medical Institute, Divisions of Human Biology and Public Health Sciences, Fred Hutchinson Cancer Research Center, Seattle, Washington, United States of America; 2 Biochemistry, Molecular Biology, and Biophysics Department, University of Minnesota Medical School, Minneapolis, Minnesota, United States of America; The University of North Carolina at Chapel Hill, UNITED STATES

## Abstract

The Fanconi anemia (FA)-BRCA pathway mediates repair of DNA interstrand crosslinks. The FA core complex, a multi-subunit ubiquitin ligase, participates in the detection of DNA lesions and monoubiquitinates two downstream FA proteins, FANCD2 and FANCI (or the ID complex). However, the regulation of the FA core complex itself is poorly understood. Here we show that the FA core complex proteins are recruited to sites of DNA damage and form nuclear foci in S and G2 phases of the cell cycle. ATR kinase activity, an intact FA core complex and FANCM-FAAP24 were crucial for this recruitment. Surprisingly, FANCI, but not its partner FANCD2, was needed for efficient FA core complex foci formation. Monoubiquitination or ATR-dependent phosphorylation of FANCI were not required for the FA core complex recruitment, but FANCI deubiquitination by USP1 was. Additionally, BRCA1 was required for efficient FA core complex foci formation. These findings indicate that FANCI functions upstream of FA core complex recruitment independently of FANCD2, and alter the current view of the FA-BRCA pathway.

## Author Summary

Fanconi anemia is a genetic disease characterized by bone marrow failure, congenital malformations and cancer predisposition. Cells derived from Fanconi anemia patients have a dysfunctional FA-BRCA pathway and are deficient in the repair of a specific form of DNA damage, DNA interstrand-crosslinks, that are induced by certain chemotherapeutic drugs. Therefore, the study of FA-BRCA pathway regulation is essential for developing new treatments for Fanconi anemia patients and cancer patients in general. One of the first steps in the pathway is the detection of DNA lesions by the FA core complex. We have optimized a method to visualize the recruitment of the FA core complex to sites of DNA damage and, for the first time, explored how this process occurs. We have uncovered several factors that are required for this recruitment. Among them is a FA core complex substrate, FANCI. We report that non-phosphorylated FANCI, previously believed to be an inactive form, has an important role in the recruitment of the FA core complex and DNA interstrand-crosslink repair. Our findings change the current view of the FA-BRCA pathway and have implications for potential clinical strategies aimed at activating or inhibiting the FA-BRCA pathway.

## Introduction

Fanconi anemia (FA) is a rare genetic disorder characterized by bone marrow failure, congenital malformations and cancer susceptibility [1]. Eighteen FA genes have been identified (*FANC-A*, -*B*, -*C*, -*D1/BRCA2*, -*D2*, -*E*, -*F*, -*G*, -*I*, -*J/BRIP1*, -*L*, -*M*, -*N/PALB2*, -*O/RAD51C*, -*P/SLX4*, -*Q/XPF*, -*S/BRCA1* and -*T/UBE2T*) [2–9]. The FA proteins function in a common DNA repair pathway (the FA pathway or the FA-BRCA pathway), which coordinates the repair of interstrand-crosslinks (ICLs). Disruption of this pathway renders cells sensitive to ICL-inducing agents, such as mitomycin C (MMC) [10]. Several FA genes are also breast and ovarian cancer susceptibility genes (*FANCD1/BRCA2*, *FANCJ/BRIP1*, *FANCN/PALB2*, *FANCO/RAD51C*, and *FANCS/BRCA1*). Among these, BRCA2, PALB2, RAD51C and BRCA1 have well-defined roles in homologous recombination (HR), linking the FA pathway to HR-mediated repair [11–13].

Eight of the FA proteins (FANC-A, -B, -C, -E, -F, -G, -L and -M) form a ubiquitin ligase complex (the FA core complex) with other associated proteins (FAAP-10/MHF2, -16/MHF1, -20, -24 and -100) in the nucleus [14–16]. Among the FA core complex subunits, FANCM, in complex with FAAP24, is a platform for recruiting the rest of the FA core complex to chromatin [17, 18]. The FA core complex is involved in sensing the DNA lesions and monoubiquitinates FANCD2 and FANCI [14–16, 19]. Monoubiquitination of FANCD2 and FANCI is required for their localization to sites of DNA damage and efficient ICL-repair [20, 21]. FANCD2 and FANCI work together as a protein complex (the ID complex) [21, 22]. Optimal monoubiquitination of FANCD2 and FANCI also requires the ATR-dependent phosphorylation of FANCI at the S/TQ cluster domain [23, 24].

Many proteins involved in DNA repair, including several FA proteins (FANCD2, FANCI, FANCJ, BRCA2, BRCA1, PALB2, SLX4, XPF and BRCA1), accumulate at sites of DNA damage. This accumulation can be visualized as distinct nuclear foci. Visualization of foci has been a fundamental technique for understanding DNA repair pathways: (i) uncovering new players, (ii) identifying sequential steps in the pathways, (iii) understanding the interplay between different DNA repair pathways, and (iv) identifying mechanisms of regulation [25]. However, the recruitment of the FA core complex is poorly understood, due to the difficulty of detecting FA core complex proteins as nuclear foci, although FA core complex accumulation at sites of DNA damage has been sporadically reported [26–28]. To address this, we have optimized the immunocytochemical detection method, allowing the visualization of the FA core complex foci. This enabled us, for the first time, to comprehensively analyze how this early process in the activation of the FA pathway is regulated. Surprisingly, we have found that FANCI, which has been shown to work downstream of the FA core complex, is also required for efficient accumulation of the FA core complex at sites of DNA damage. This FANCI function was independent of its binding partner FANCD2, FANCI monoubiquitination and FANCI phosphorylation. USP1 regulated FA core complex foci formation by deubiquitinating FANCI. Additionally, BRCA1 was required for efficient FA core complex foci formation. Our work challenges the linear, canonical model of the FA-BRCA pathway, and expands on the mechanism of its activation.

## Results

### FA core complex proteins accumulate at sites of DNA damage in a manner dependent on the whole FA core complex

**Fig 1:**
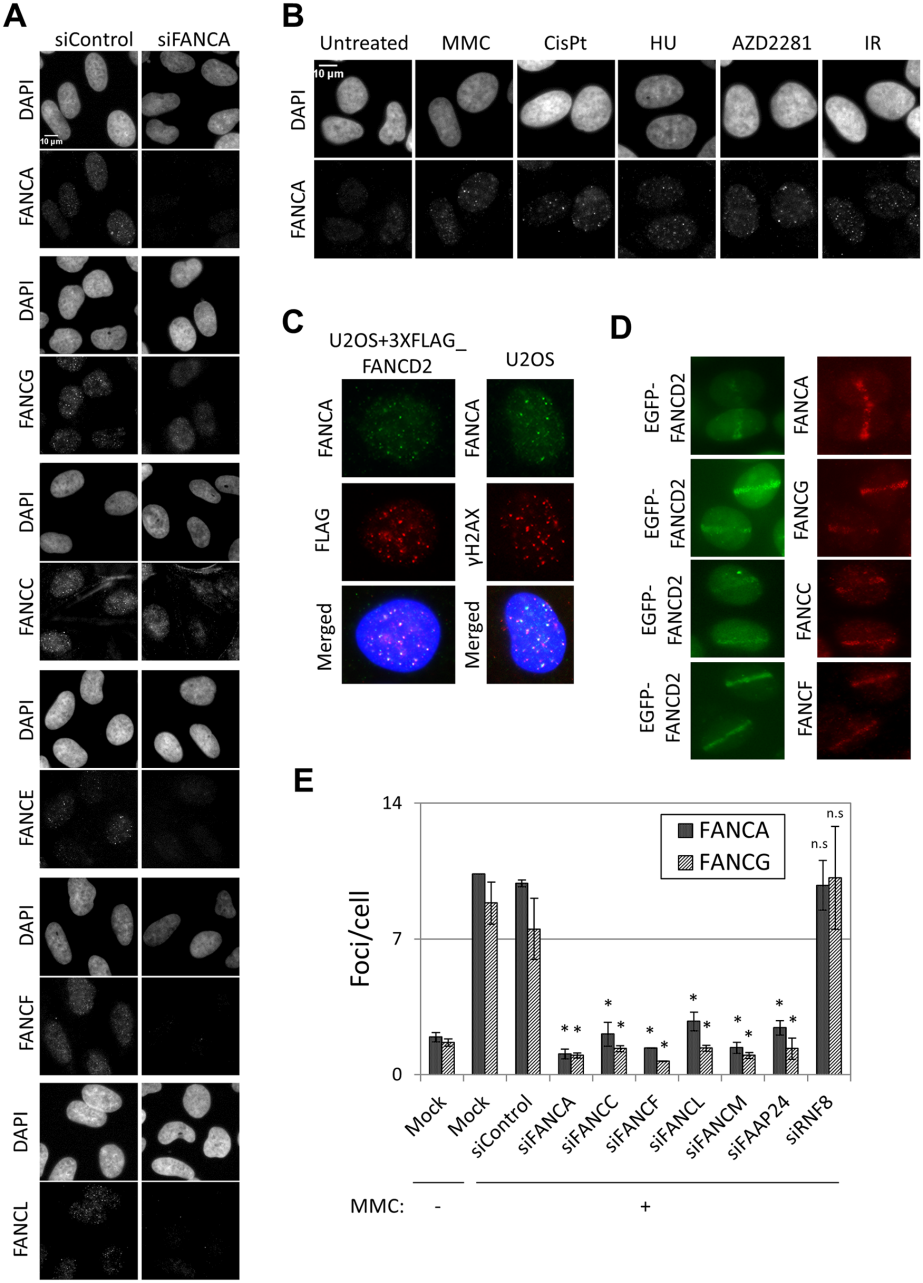
FA core complex forms foci at sites of DNA damage. (A) U2OS cells were transfected with siControl or siFANCA and treated with mitomycin C (MMC) 60ng/ml for 24h before fixation. Cells were immunostained with the indicated antibodies. (B) Cells were left untreated or treated with 60ng/ml MMC, 2.5 μM cisplatin, 250μM hydroxyurea (HU) or 10μM AZD2281 for 24h, or treated with 10Gy ionizing radiation (IR) 8h before fixation. Then cells were immunostained with anti-FANCA antibody. Representative images are shown. (C) U2OS cells or U2OS expressing 3xFLAG-FANCD2 were treated with MMC 60ng/ml for 24h and immunostained with anti-FANCA, FLAG and γH2AX antibodies. (D) Localized DNA damage induced with a 450nm laser in U2OS cells expressing EGFP-FANCD2. Cells were fixed 30min after irradiation and immunostained with the indicated antibodies. (E) U2OS cells were transfected with the indicated siRNAs, untreated or treated with MMC 60ng/ml for 24h and immunostained with anti-FANCA or anti-FANCG antibodies. Foci/cell were counted using an automated software. Data represent mean values ± SD of three independent experiments. (*) Indicates p < 0.05; (n.s.) indicates no statistical significance (Compared to siControl).

We carefully optimized immunostaining methods (described in Methods) and successfully detected nuclear foci of FANCA, FANCC, FANCE, FANCF, FANCG and FANCL in U2OS cells treated with MMC (Fig 1A). All of these foci were reduced in FANCA-depleted cells, suggesting that foci formation of the FA core complex proteins is FANCA-dependent and are likely to represent foci formation of the canonical FA core complex. Specificity of the antibodies was also confirmed by western blotting (S1G, S2E, S3C and S3D Figs). The formation of FANCA, FANCG and FANCE foci was induced by treatment with various DNA damaging agents (MMC, cisplatin, hydroxyurea, a PARP inhibitor (AZD2281) and ionizing radiation (IR)), in U2OS cells (Fig 1B and S1A Fig). FANCA and FANCG foci were also observed in HeLa cells and fibroblasts (S1B, S1C and S1E–S1G Fig). FANCA substantially colocalized with FANCD2 and γH2AX, but not TRF1, suggesting that FA core complex proteins localize at sites of DNA damage and not at telomeres (Fig 1C and S1D Fig). Recruitment of FANCA, FANCG, FANCC and FANCF to laser-induced localized DNA damage was also detected (Fig 1D). We were also successful in detecting foci formation of exogenously expressed myc-tagged FANCG (S4 Fig). In this case, detection of the foci was dependent on the level of expression. While high FANCG expression (pMMP-FANCG construct) resulted in pan-nuclear FANCG staining, foci were clearly detected (both with antibodies against FANCG and MYC-tag) when a low expressing (pLentiX1-mycFANCG) was used (S4A and S4B Fig). Both high-expression and low-expression FANCG constructs rescued MMC sensitivity at the same level (S4C Fig).

The fact that all FA core complex proteins we tested formed foci at sites of DNA damage and that their recruitment was dependent on FANCA (Fig 1A) suggests that these proteins are present at sites of DNA damage as part of the FA core complex. To test this further, we examined the effects of depleting other components of the FA core complex on FA core complex foci formation. Depletion of FANCA, FANCC, FANCF or FANCL abolished the formation of FANCA, FANCG, FANCC, FANCL and FANCD2 foci, but not γ-H2AX foci (Fig 1E and S2A–S2C Fig). Similarly, formation of FANCA and FANCG foci was impaired in FANCF-deficient cells (TOV21G), but was restored in the corrected cells (S2D and S2E Fig). The FA core complex is loaded onto chromatin through its interaction with FANCM/FAAP24 [17]. RNF8 has also been reported to mediate the recruitment of the FA core complex to psoralen- and laser-induced localized DNA damage [29]. FANCM and FAAP24 depletion abrogated the formation of FANCA, FANCG, FANCC and FANCL foci after MMC without affecting their protein levels (Fig 1E and S2A, S3A and S3B Figs). On the other hand, RNF8 depletion did not affect FANCA, FANCG, FANCC, FANCL or FANCD2 foci, while BRCA1 foci were reduced (Fig 1E and S2A, S2B, S2F and S2G Fig), consistent with the previous reports [30]. These findings suggest that the whole FA core complex including FANCM/FAAP24, but not RNF8, is critical for recruitment of the FA core complex to sites of DNA damage.

### FA core complex foci form in S and G2 phases of the cell cycle

**Fig 2:**
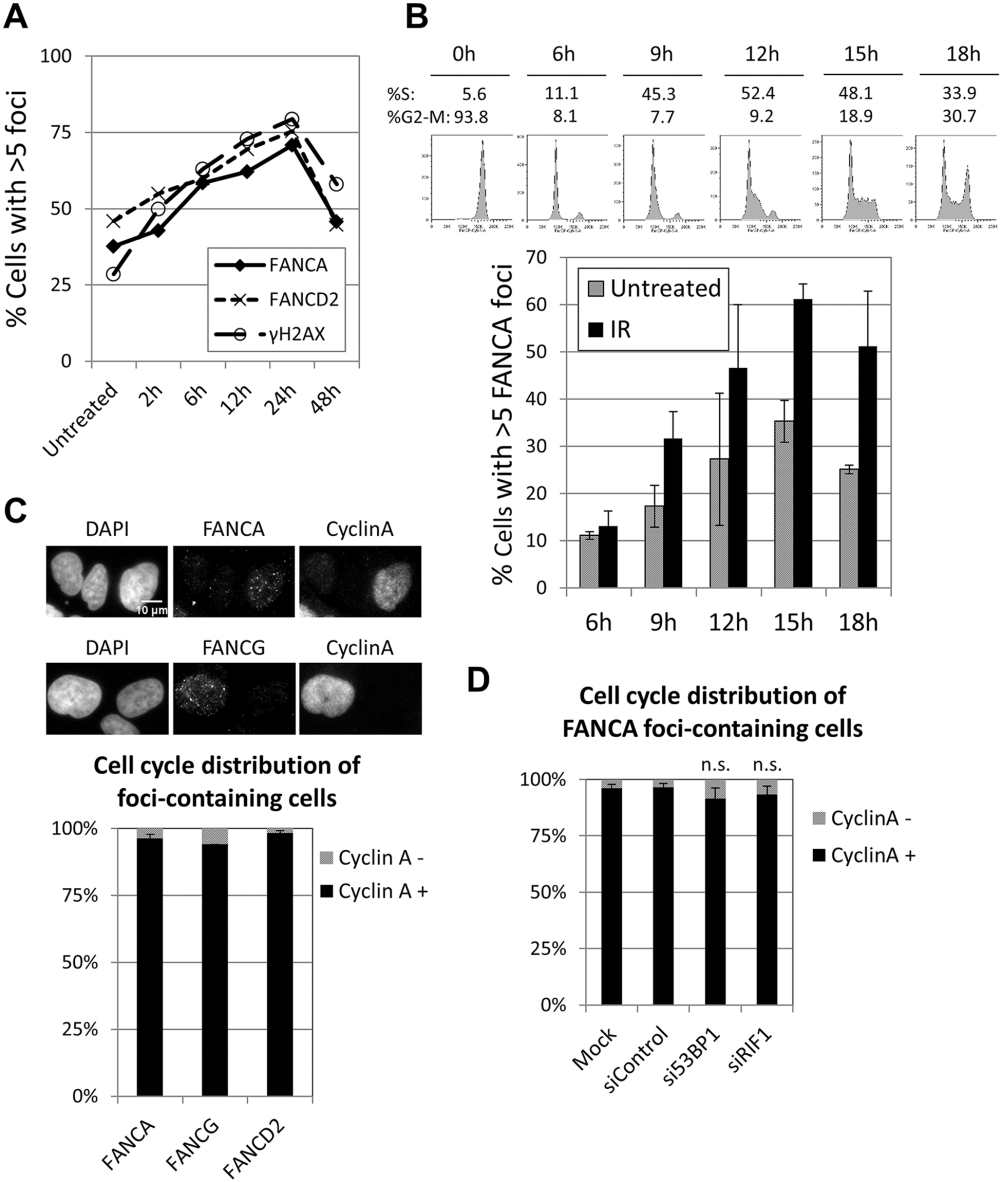
FA core complex foci form during S-G2 phases of the cell cycle. (A) U2OS cells were treated with MMC for 1h at 60ng/ml, then released into fresh media and fixed at the indicated time points. Cells were immunostained with the indicated antibodies and quantified. The percentage of cells with more than 5 foci is shown. (B) Cells were arrested in M-phase with nocodazole, re-seeded in fresh media and collected at the indicated times. The upper panel shows cell cycle profiles (PI staining). The lower panel shows percentage of cells with more than 5 FANCA foci (n = 3, mean ± SD). Cells were untreated or irradiated with 10Gy IR 1h before each time point. (C) Cells were irradiated with 10Gy IR 2h before fixation. Graph shows percentage of cyclin A-positive and -negative cells in the FANCA-, FANCG- or FANCD2-foci containing cells population (n = 3, mean ± SD). Upper panel shows representative images. (D) Cells were transfected with indicated siRNAs and irradiated with 10Gy IR 2h before fixation. Graph shows percentage of cyclin A-positive and -negative cells in the FANCA-foci containing cells (n = 3, mean ± SD).

The foci formation kinetics of FA core complex was similar to that of FANCD2, both after MMC pulse treatment (Fig 2A) and IR exposure (S5A Fig). Cell synchronization after release from nocodazole arrest revealed that FANCA foci were efficiently induced in S and G2 phases, in untreated cells or after IR, but not in G1 (Fig 2B). Furthermore, more than 95% of cells with FANCA or FANCG foci were cyclin A (a S/G2-phase marker)-positive (Fig 2C). These data indicate that FA core complex foci form during S and G2 phases. Similar results were obtained for FANCD2 foci (Fig 2C).

BRCA1, which normally forms foci in S and G2 phases, is able to localize to sites of DNA damage during G1 when 53BP1 or RIF1 are absent [31, 32]. Consistent with these reports, the proportion of BRCA1 foci-containing cells was vastly greater than the proportion of cyclin A-positive cells when 53BP1 or RIF1 were depleted (S5C and S5D Fig), demonstrating that BRCA1 foci formed in G1 in these conditions. In contrast, depletion of 53BP1 or RIF1 did not significantly alter the cell cycle distribution of FANCA (or FANCD2) foci-containing cells, with more than 90% of them corresponding to cyclin A-positive cells (Fig 2D and S5B Fig). These results indicate that the FA core complex and FANCD2 foci form almost exclusively in S and G2, even in the absence of 53BP1 or RIF1, and suggest that the mechanisms of recruitment are distinct from those of BRCA1.

### ATR is required for FA core complex foci formation

**Fig 3:**
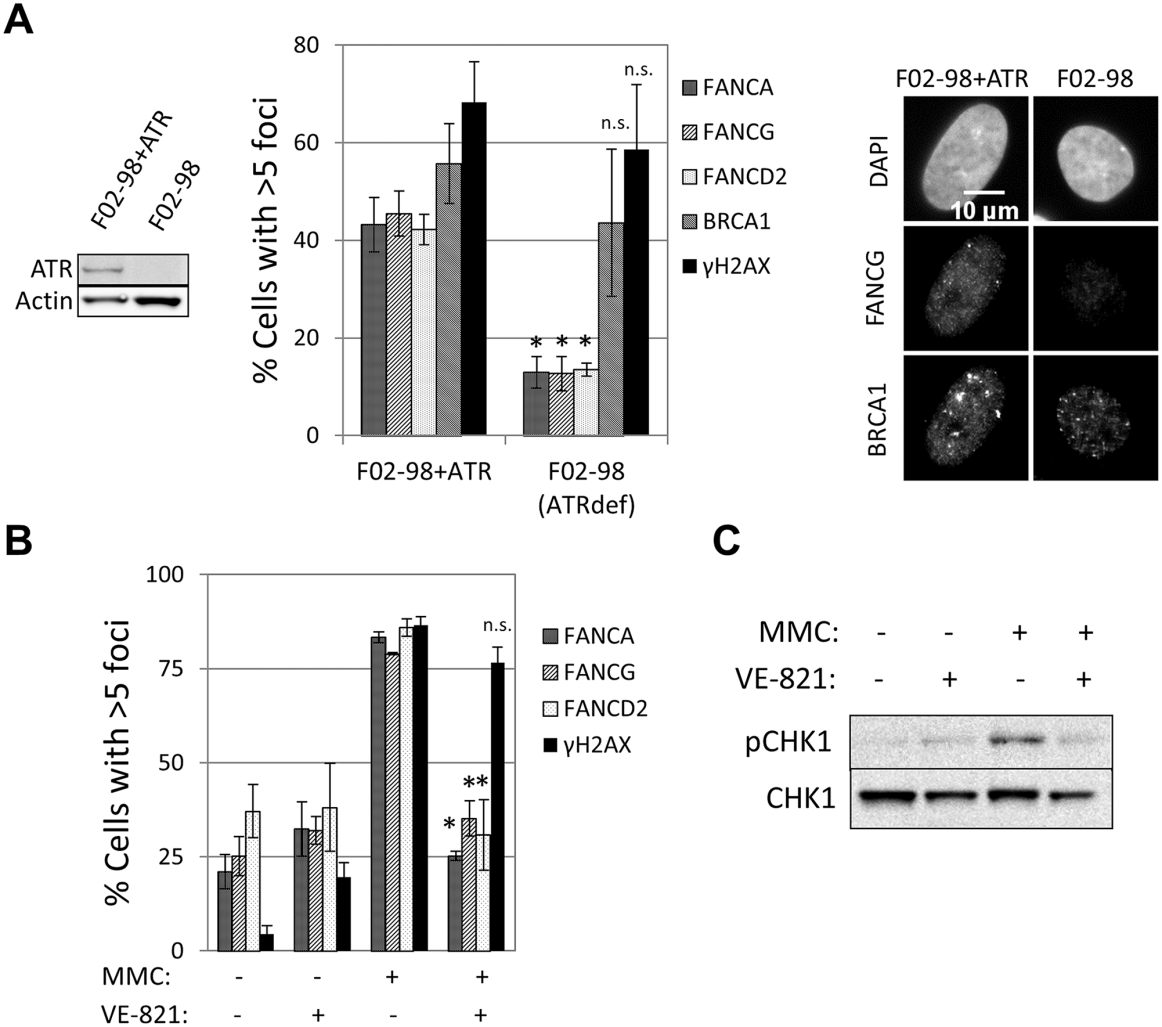
ATR is required for FA core complex foci formation. (A) ATR deficient F02-98tert cells and complemented cells were treated with 60ng/ml MMC for 24h before fixation and staining. Percentage of cells with >5 foci (n = 3, mean ± SD) and representative images are shown. (*) Indicates p<0.05; (n.s.) indicates no statistical significance. The left panel shows ATR protein expression in these cell lines (western blotting). (B) U2OS cells were pretreated with 10μM VE-821 for 2h and then treated with MMC (60ng/ml) for 24h in the presence of VE-821. Then, cells were fixed and immunostained with the indicated antibodies. Percentage of cells with >5 foci is shown (n = 3, mean ± SD). (*) Indicates p<0.05; (n.s.) indicates no statistical significance (Compared to “MMC treated—no VE-821” sample). (C) Cells were treated with the same conditions as in (B). pCHK1 (Serine 345) western blotting confirmed ATR inhibition by VE-821.

The ability to detect recruitment of FA core complex proteins at sites of DNA damage allowed us to search for factors required for the formation of these foci and therefore gain deeper insight into how the FA pathway is regulated. ATR is the primary kinase that controls FA pathway activation [23, 24, 33]. However, whether ATR is required for FA core complex recruitment is unknown. A strong reduction in the number of cells containing FANCA, FANCG, FANCC, FANCL or FANCD2 foci was observed in the ATR-deficient cells (F02-98 fibroblasts and ATR-depleted U2OS cells), but not in ATR-proficient control cells (Fig 3A and S6A and S6B Fig). ATR specific inhibitor (VE-821) [34], but not ATM inhibitor, impaired the formation of both FA core complex and FANCD2 foci (Fig 3B and S3C, S6C and S6D Fig), indicating that ATR kinase activity is required for FA core complex foci formation.

### FANCI, but not FANCD2, is required for FA core complex foci formation

**Fig 4:**
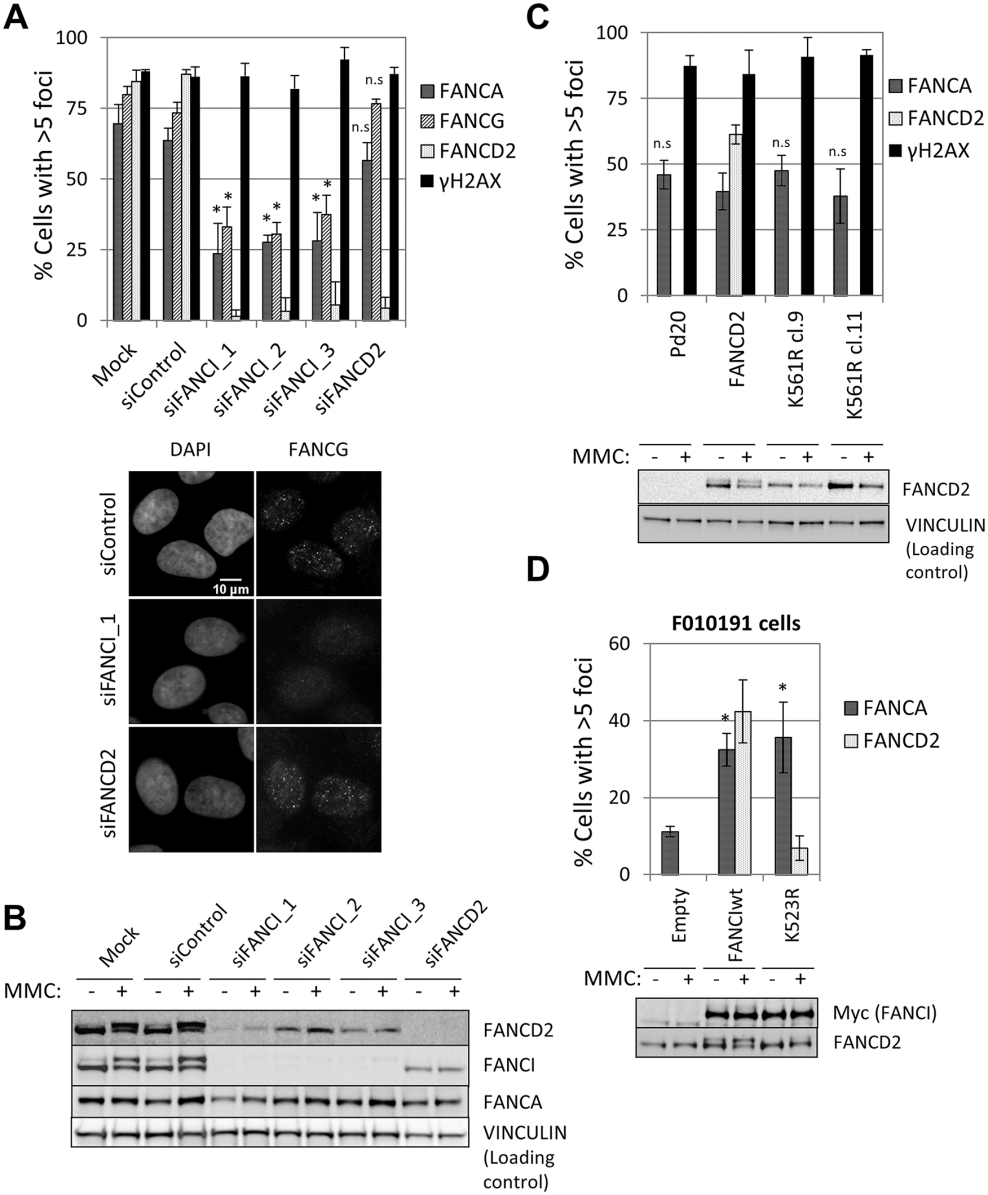
FANCI, but not FANCD2, is required for FA core complex foci formation. (A) U2OS cells were transfected with the indicated siRNAs and treated with MMC (60 ng/ml) for 24 hours. Cells with >5 foci were counted and the percentage of positive cells is shown in upper panel (n = 3, mean ± SD). (*) Indicates p < 0.05; (n.s.) indicates no statistical significance. Representative images are shown. (B) Western blotting. U2OS cells transfected with the indicated siRNAs and treated with MMC (60 ng/ml) for 24 hours. (C) FANCD2-deficient PD20 fibroblasts and PD20 fibroblasts complemented with the indicated FANCD2 constructs were treated with MMC (60 ng/ml) for 24 hours. The percentage of foci positive-cells is shown (n = 3, mean ± SD). (*) Indicates p<0.05; (n.s.) indicates no statistical significance (Compared to PD20+FANCD2). Immunoblot analyses showed FANCD2 expression and ubiquitination status. (D) FANCI-deficient F010191 fibroblasts were transduced with FANCI constructs (wild-type or K523R mutant). The percentage of foci positive-cells after treatment with MMC (60 ng/ml) for 24h is shown (n = 3, mean ± SD). (*) Indicates p<0.05 (Compared to F010191-Empty). Immunoblot analyses showed FANCI expression and ubiquitination status.

FANCD2 and FANCI function together downstream of the FA core complex [21, 22]. Unexpectedly, we observed a decrease in FANCA, FANCG, FANCC and FANCL foci formation when FANCI, but not FANCD2, was depleted in U2OS cells (Fig 4A and 4B and S7 Fig), suggesting that only FANCI is required for efficient FA core complex foci formation. Consistent with this, FANCD2-deficient fibroblasts (PD20) and PD20 transfected with wild-type FANCD2 or non-ubiquitinatable K561R mutant of FANCD2 showed similar degrees of FANCA foci formation (Fig 4C).

In the absence of FANCD2, FANCI was not ubiquitinated (Fig 4B) and was not bound to chromatin (S10C Fig), suggesting that FANCI ubiquitination and chromatin binding are dispensable for FA core complex foci formation. In a FANCI-deficient fibroblast cell line F010191 [22], wild-type FANCI, but not a non-ubiquitinatable K523R mutant or a DNA-binding mutant K294E/K339E [35], were able to sustain FANCD2 foci (Fig 4D and S7B Fig), as previously described [36]. In contrast, the three FANCI constructs (wild-type, K523R and K294E/K339E) rescued FANCA foci (Fig 4D and S7B–S7D Fig), indicating that FANCI is required for FA core complex foci independently of its ubiquitination and DNA binding.

### FANCI promotes FA core complex foci formation independently of ATR

**Fig 5:**
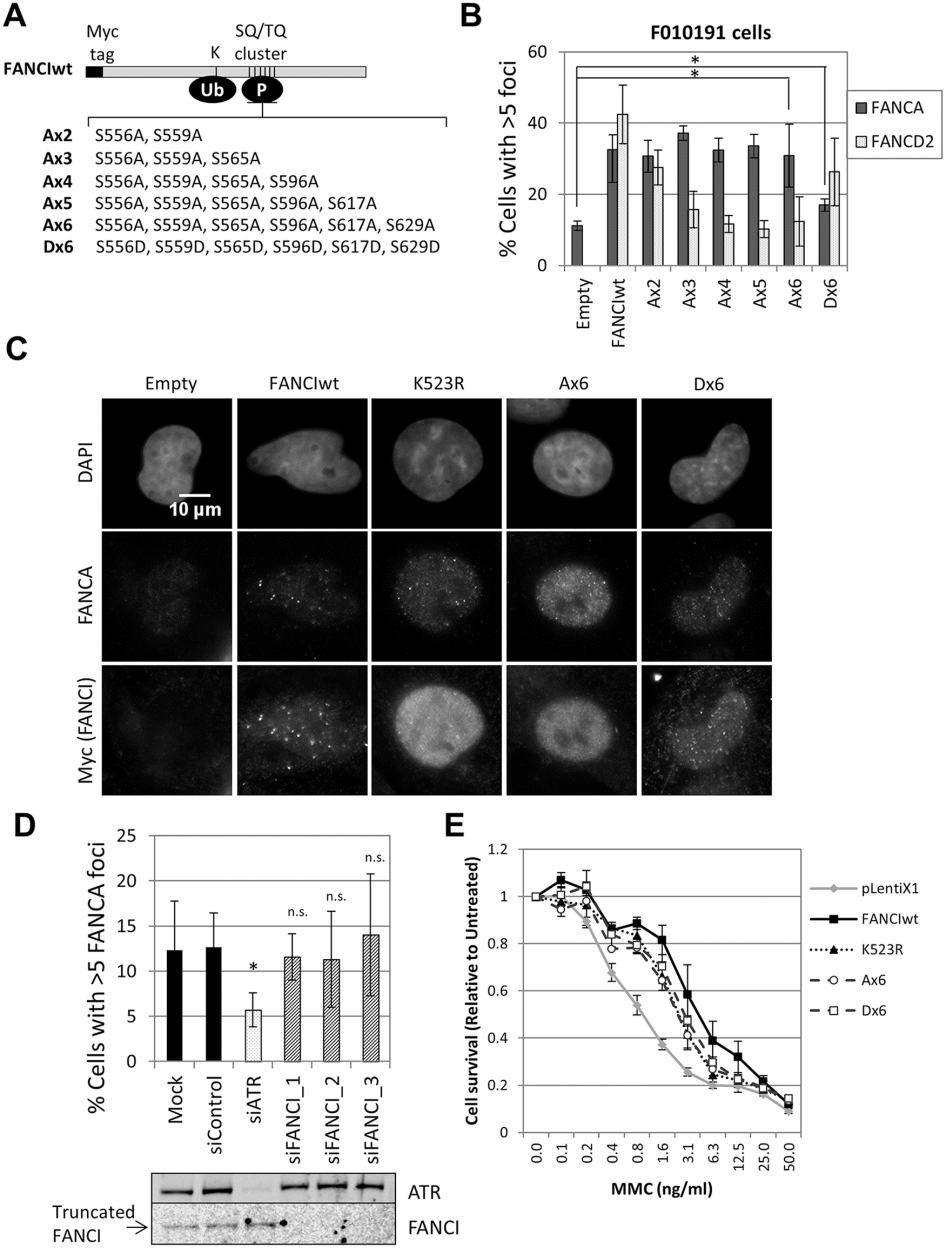
FANCI phosphorylation is not required for FA core complex foci formation. (A) A diagram of FANCI showing important domains and mutant forms analyzed: phosphorylation mutants (Ax2—Ax6) and the phosphomimetic mutant (Dx6). (B) FANCI-deficient F010191 cells were transduced with wild-type and mutant forms of FANCI. Cells were treated with MMC (60 ng/ml) for 24h. The percentage of foci-positive cells is shown (n = 3, mean ± SD). (*) Indicates p < 0.05. (C) Representative images corresponding to the experiment quantified in panel B. (D) FANCI-deficient F010191 cells were transfected with the indicated siRNAs and treated with MMC (60 ng/ml) for 24h. Percentage of foci-positive cells is shown (n = 3, mean ± SD). (*) Indicates p < 0.05; (n.s.) indicates no statistical significance. Protein depletion by siRNA was confirmed by immunoblotting (lower panel). (E) FANCI-deficient F010191 cells were transduced with wild-type and mutant forms of FANCI, plated at low density and treated with increasing concentrations of MMC. The cell-surviving fraction after 7 days, compared to untreated cells is shown (n = 3, mean ± SD).

Next we tested if ATR-mediated phosphorylation of FANCI is required for FANCA foci formation. FANCI-deficient F010191 fibroblasts were transduced with wild-type FANCI, a series of FANCI phosphomutants (Ax2-Ax6) or a phosphomimetic mutant (Dx6) (Fig 5A). Consistent with a previous report [24], increasing the number of mutated phosphorylation sites (Ax2-Ax6) resulted in progressively stronger suppression of FANCD2 foci formation and monoubiquitination (Fig 5B and S8 Fig). Expression of the Dx6 mutant resulted in increased basal FANCD2 and FANCI ubiquitination and partial restoration of FANCD2 foci (Fig 5B and S8 Fig). In contrast, all FANCI phosphomutants were able to rescue FANCA foci, while the Dx6 mutant rescued FANCA foci partially (Fig 5B and 5C). Similar results were obtained using FANCI-knockout HCT116 cells (S9 Fig). In this cell line, however, transduction of the Dx6 mutant resulted in a full correction of FANCA foci. These results demonstrate that FANCI promotes FA core complex foci formation independently of the phosphorylation status of the S/TQ cluster domain.

ATR could also promote FA core complex foci formation through FANCI phosphorylation at other sites outside the S/TQ cluster domain. To test if ATR and FANCI promote FA core complex foci formation through shared or distinct mechanisms, we depleted ATR in FANCI-deficient cells (F010191). Compared to FANCI deficiency only, ATR depletion resulted in an additional defect in FANCA foci formation (Fig 5D), suggesting that ATR and FANCI promote FA core complex recruitment through independent mechanisms. Identical results were obtained when ATR and FANCI were co-depleted in U2OS (S10A Fig). F010191 cells express a C-terminal truncated form of FANCI [22, 36]. Depletion of this truncated FANCI did not further suppress FANCA foci, indicating that the truncated FANCI does not support FA core complex foci formation (Fig 5D).

To better understand how ATR and FANCI promote recruitment of the FA core complex to sites of DNA damage, we analyzed the binding of FANCA, FANCC and FANCL to chromatin using cell fractionation. Relative to untreated cells, the amount of chromatin-bound FANCA, FANCC or FANCL was not increased in MMC-treated cells (S10D Fig), indicating that FA core complex chromatin binding and foci formation are two discrete steps. In U2OS cells, FANCI depletion, but not ATR or FANCD2 depletion, significantly reduced the amount of FANCA bound to chromatin (insoluble fraction) (S10B and S10C Fig). This data further supports the previous observations that FANCI regulates FA core complex recruitment at sites of DNA damage in a FANCD2- independent manner, and through a mechanism distinct from ATR-mediated recruitment. However, the same defect in FANCA chromatin binding was not observed when comparing FANCI-knockout and wild-type HCT116 (S10E Fig), although depletion of FANCM did not cause the expected reduction of chromatin-bound FANCA either. The cause of discrepancy between the two cell lines is not obvious. It suggests that FANCI may facilitate two different steps in the regulation of FA core complex recruitment at sites of DNA damage in a context-dependent manner: 1) binding to chromatin, and 2) accumulation at sites of DNA damage (or foci formation).

### Non-phosphorylated FANCI contributes to cellular resistance to MMC

Our data indicates that non-phosphorylated FANCI is able to perform part of its functions within the FA pathway by promoting FA core complex recruitment. Therefore, we hypothesized that non-phosphorylated FANCI may contribute to cellular resistance to ICL-inducing agents. To test this, we analyzed MMC sensitivity of FANCI-deficient F010191 cells transduced with wild-type, K523R, Ax6 or Dx6 FANCI constructs. Consistent with our hypothesis, K523R, Ax6 and Dx6 mutants partially rescued MMC resistance, at similar levels (Fig 5E). Similar results were obtained using FANCI-knockout HCT116 cells (S9D Fig). This suggests that FA core complex foci formation may play a role in conferring cellular resistance to ICL-inducing agents.

### BRCA1 and USP1 are required for FA core complex foci formation

Our demonstration of a role for FANCI upstream of FA core complex recruitment suggests that the FA pathway may not be as linear as current models suggest. It also raises the possibility that other factors known to work downstream of the FA core complex may also regulate FA core complex recruitment. To test this, we analyzed FA core complex foci formation in cells deficient in several other proteins involved in the FA pathway (BRCA1/FANCS, BRCA2/FANCD1, CtIP, FANCJ/BRIP1, FANCN/PALB2, FANCP/SLX4, FANCQ/XPF and USP1) (S2 Table). Surprisingly, both BRCA1 and USP1 were required for FANCA and FANCG foci formation.

**Fig 6:**
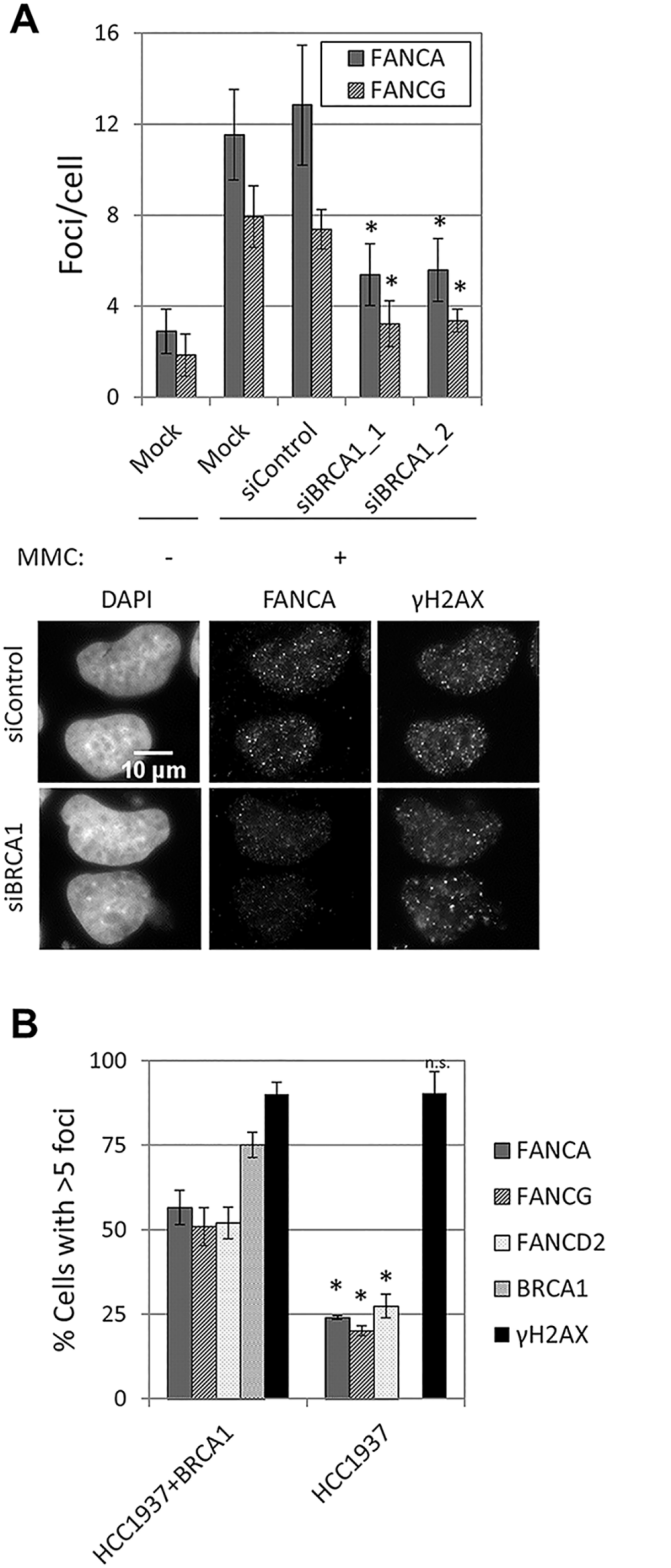
BRCA1 is required for FA core complex foci formation. (A) U2OS cells were transfected with the indicated siRNAs, left untreated or treated with MMC (60 ng/ml) for 24h. The number of foci/cell (n = 3, mean ± SD) and representative images are shown. (*) Indicates p<0.05 (Compared to siControl for each foci, respectively). (B) BRCA1-deficient HCC1937 and complemented cell lines were treated with MMC (60 ng/ml) for 24h. The percentage of cells with > 5 foci (n = 3, mean ± SD) is shown. (*) Indicates p<0.05; (n.s.) indicates no statistical significance (Compared to HCC1937+BRCA1 for each foci, respectively).

Depletion of BRCA1 resulted in a strong reduction in FANCA, FANCG, FANCC, FANCL and FANCD2 foci formation in U2OS and HeLa cells (Fig 6A and S11A and S11B Fig), without affecting FANCA protein levels or FANCD2 monoubiquitination (S11C Fig). A similar reduction in FA core complex foci-containing cells was observed in BRCA1-deficient HCC1937, when compared to BRCA1-complemented HCC1937 (Fig 6B). To better characterize how BRCA1 promotes FA core complex foci formation, we then tested whether this function was mediated by known BRCA1-interacting proteins (S2 Table and S11D–S11H Fig). No defect in FANCA or FANCG foci was observed in cells deficient in FANCJ/BRIP1, FANCD1/BRCA2, FANCN/PALB2, FAM175A/ABRAXAS, UIMC1/RAP80 or RBBP8/CtIP, suggesting that BRCA1 performs this function independently, or through a binding partner other than the ones tested.

**Fig 7:**
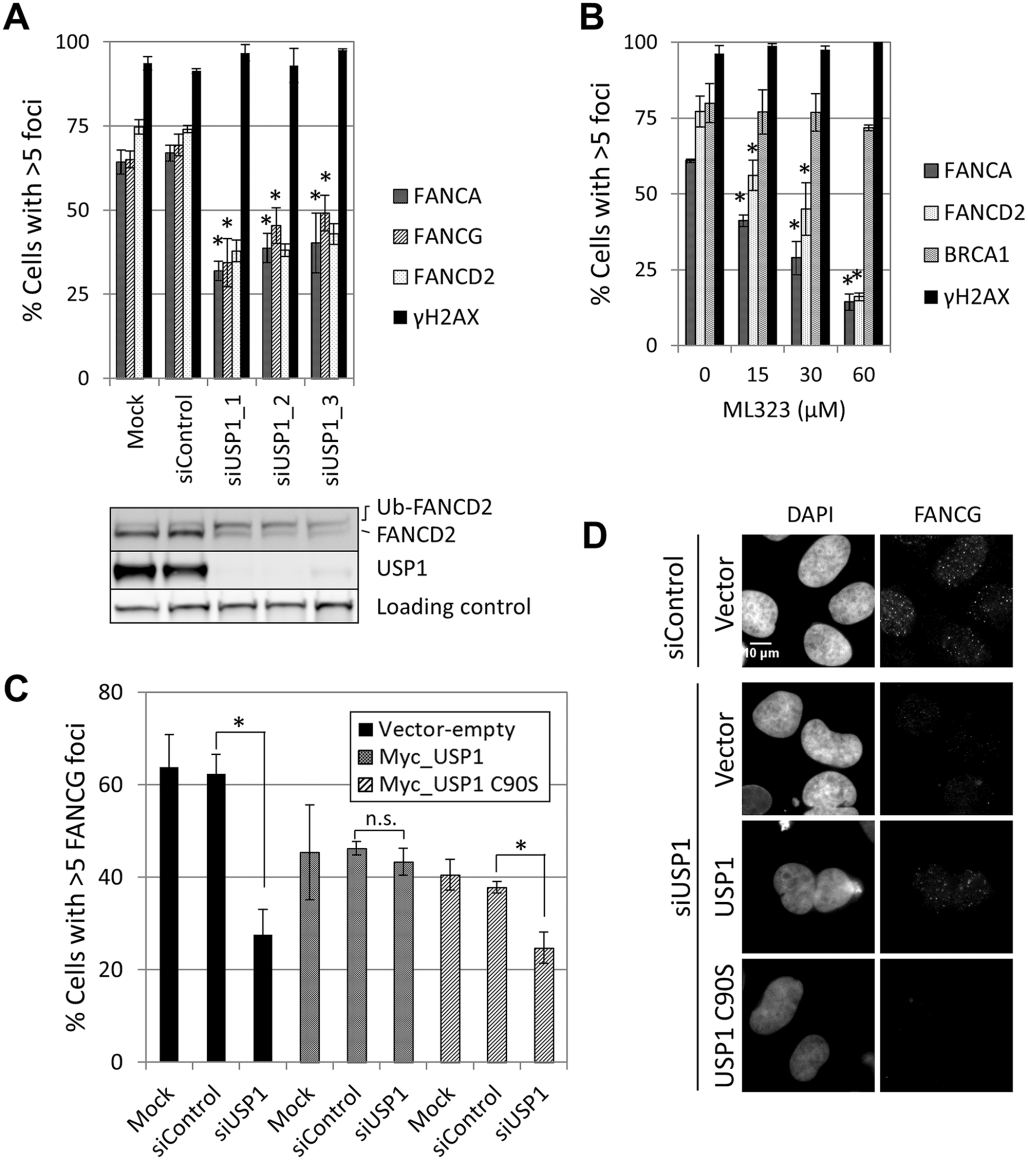
USP1 is required for FA core complex foci formation. (A) U2OS cells were transfected with the indicated siRNAs and treated with 10Gy IR. The cells were fixed and stained 8 hours later. Cells with > 5 foci were counted and the percentage of positive cells is shown (n = 3, mean ± SD). (*) Indicates p<0.05; (Compared to siControl for each foci, respectively). The lower panel shows a western blot corresponding to samples shown in upper panel. (B) Cells were pre-treated with ML323 at the indicated dose 2 hours before irradiation (10 Gy IR). Cells were fixed and stained 8 hours later. Graph shows cells containing >5 foci (n = 3, mean ± SD). (*) Indicates p<0.05; (Compared to Untreated sample for each foci, respectively). (C) Cells were transfected with the indicated combinations of siRNA and plasmid 48 hours before treatment (IR 10 Gy, fixed 8 hours later). The percentage of cells containing > 5 FANCG foci (n = 3, mean ± SD) is shown. (*) Indicates p<0.05. (n.s.) indicates no statistical significance. In samples transfected with USP1 wild-type or USP1 C90S, only USP1-expressing cells (Myc-positive) were included in the analysis. (D) Representative images corresponding to the experiment detailed in panel C.

FANCA,FANCG, FANCC and FANCL foci formation were impaired in USP1-depleted U2OS cells and in cells treated with a USP1 specific inhibitor, ML323 [37] (Fig 7A and 7B and S12A Fig). Increasing concentrations of ML323 resulted in decreased FANCA and FANCD2 foci, without affecting BRCA1 or γH2AX foci (Fig 7B). ML323 treatment resulted in increased FANCD2 and FANCI ubiquitination, confirming USP1 inhibition (S12B Fig). To confirm the siRNA and inhibitor data, USP1-depleted cells were complemented using an siRNA-resistant USP1 cDNA. Due to the high instability of overexpressed wild-type USP1 protein, a form of USP1 that is unable to cleave itself, GG670/671AA, (described in [38]) was used. The catalytic function of GG670/671AA mutant of USP1 is comparable to wild-type USP1 [38]. The FA core complex and FANCD2 foci defect was rescued when wild-type USP1, but not the catalytic inactive form (USP1 C90S), was expressed (Fig 7C and 7D and S12C Fig). In both cases, an overall reduction in the number of cells with foci was observed, likely due to cellular toxicity of USP1 overexpression. These data indicate that catalytic activity of USP1 is required to promote efficient recruitment of FA core complex and FANCD2 to sites of DNA damage. They also indicate that impaired FA core complex recruitment at sites of DNA damage does not necessarily translate into deficient FANCD2-FANCI ubiquitination.

### Deubiquitination of FANCI by USP1 is required for FA core complex foci formation

**Fig 8:**
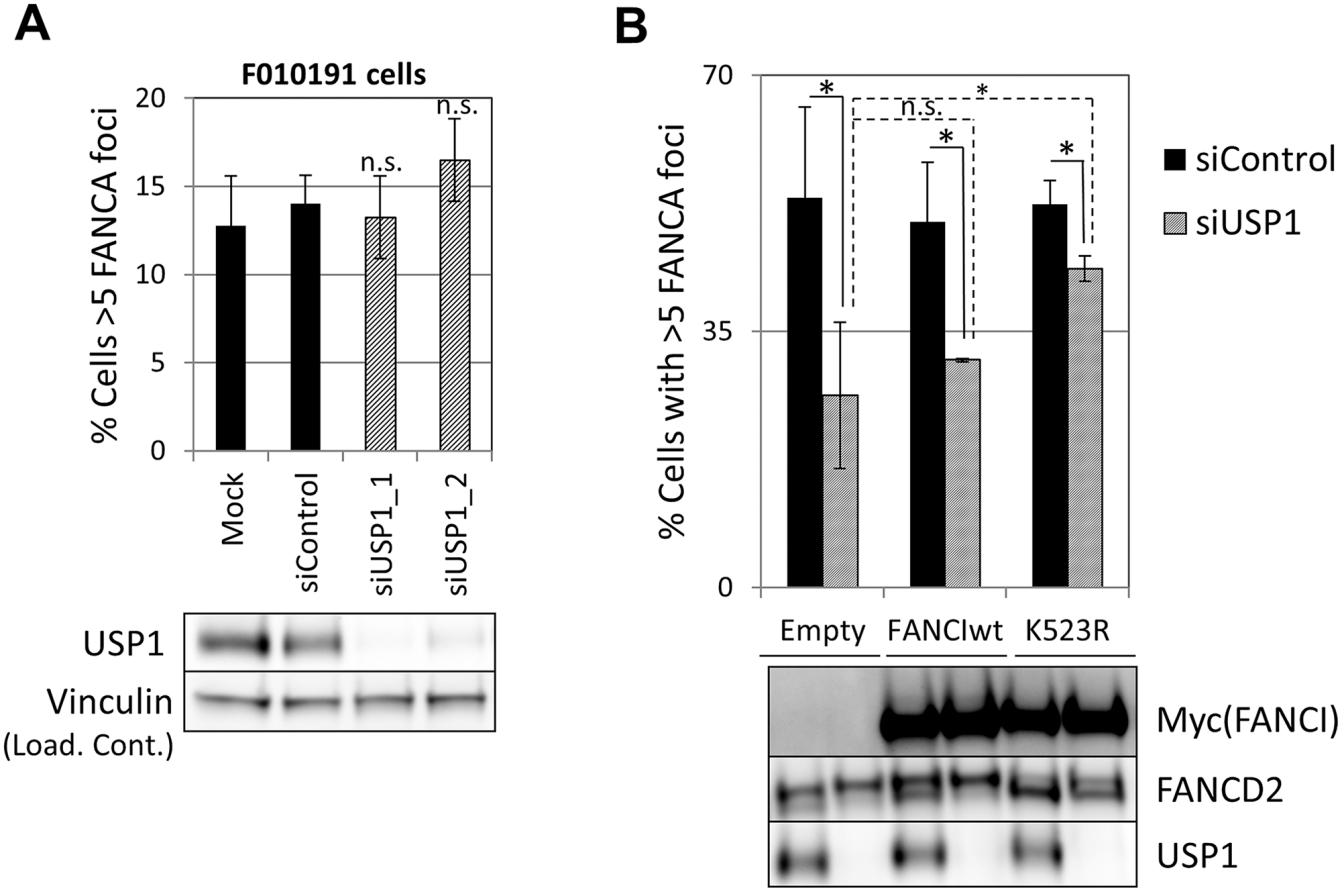
Deubiquitination of FANCI by USP1 is required for FA core complex foci formation. (A) FANCI-deficient F010191 cells were transfected with the indicated siRNAs, treated with MMC and then fixed and stained with FANCA antibody. Percentage of cells containing 5 or more foci is shown (n = 3, mean ± SD). (n.s.) indicates no statistical significance. Protein extracts were subjected to western blotting to confirm depletion of USP1 (lower panel). (B) U2OS cells transduced with the indicated constructs were transfected with the siControl or siUSP1. 48h later they were treated with MMC and then fixed and stained with FANCA antibody. Upper panel shows percentage of cells containing 5 or more foci (n = 3, mean ± SD). (*) Indicates p<0.05. (n.s.) indicates no statistical significance. Lower panel shows protein extracts from the same experiment subjected to western blotting.

Since both non-ubiquitinated FANCI and USP1 catalytic activity promoted FA core complex foci formation, next we tested the possibility that FANCI was the relevant substrate for USP1 in this function. We first tested epistasis between FANCI and USP1. USP1 was depleted using siRNA from FANCI-deficient F010191 cells. As shown in Fig 8A, USP1 depletion did not result in an increased loss of FANCA foci in FANCI-deficient cells, suggesting that FANCI and USP1 promote FA core complex foci formation through the same mechanism. If FANCI is the relevant USP1 substrate to promote FA core complex formation, overexpression of a non-ubiquitinatable FANCI should be able to rescue FA core complex foci formation in USP1-depleted cells. Therefore, we overexpressed wild-type or K523R mutant of FANCI in cells that were either transfected with siRNA control or siUSP1. As shown in Fig 8B, overexpression of the FANCI K523R mutant partially rescued FANCA foci formation in USP1-depleted cells, while the wild-type FANCI was not able to do so. These results suggest that FANCI needs to be deubiquitinated by USP1 to promote FA core complex foci efficiently.

## Discussion

Through the analyses of the FA core complex foci formation, we have elucidated several new regulatory mechanisms of the FA core complex recruitment. First, the FA core complex accumulated at sites of DNA damage in a manner dependent on the whole FA core complex, including FANCM/FAAP24, the canonical platform that loads the FA core complex to DNA [17]. An alternative mechanism of FA core complex recruitment at laser-induced localized ICLs, involving FAAP20 binding to RNF8-catalyzed polyubiquitin chains, has been described [29]. However, this mechanism did not make a significant contribution in our system. The discrepancy may be attributable to the different systems used to induce DNA damage and exemplifies the importance of assessing recruitment using foci formation.

Repair of DSBs in mammalian cells occurs mainly by two major different pathways: non-homologous end-joining (NHEJ), which predominates during G1, and homologous recombination (HR), which predominates during S and G2 [39]. As with other proteins involved in the FA pathway and HR, FA core complex foci formed during S and G2. Recently, some light has been shed on the molecular mechanisms that control DNA repair pathway choice, identifying 53BP1-RIF1 and BRCA1-CtIP as central players in this process [31, 32, 40–42]. These studies suggest that enabling BRCA1 to bind to DSBs by depleting 53BP1 or RIF1, allows for CtIP-mediated resection in G1. However, depleting 53BP1 or RIF1 was not enough to promote FA core complex or FANCD2 recruitment in G1. This data is consistent with our observation that, although efficient FA core complex recruitment depended on BRCA1, it did not depend on CtIP. Therefore, alternative activation mechanisms and/or DNA structures, not present in G1, will be needed for FA core complex recruitment at DNA lesions. Consistent with this, *in vitro* studies showed that FANCM, together with FAAP24 and MHF1-MHF2, have a strong affinity for branched DNA structures that resemble replication forks or Holliday junctions [43–45].

The recruitment of the FA core complex at sites of DNA damage is far from well understood. Our data suggests that the regulation of this process is more complex than initially envisioned. Through a candidate approach directed to proteins that participate in the repair of ICLs, we have identified four proteins that are required for FA core complex foci formation: ATR, FANCI, BRCA1 and USP1. Among these, FANCI, BRCA1 and USP1 are especially interesting, since they have been previously thought to function exclusively downstream of FA core complex. Our findings suggest that they also act upstream, by promoting FA core complex recruitment to sites of DNA damage. We show that FANCI has a function upstream of the FA core complex and independent of FANCD2. FANCD2 and FANCI were previously considered to be obligate partners: they require each other for ubiquitination, foci formation and, partially, protein stability [21, 22, 46]. More recent studies, however, have shown that FANCD2 and FANCI exhibit different responses to DNA damage [47]. Also, a FANCI-independent function of FANCD2 in promoting replication fork recovery through association with the BLMcx complex has been reported [48]. Our study supports the model that FANCD2 and FANCI have both dependent and independent roles in the DNA damage response, and identifies FA core complex foci formation as a novel FANCD2-independent function of FANCI.

Unlike FANCI function in promoting FANCD2 foci formation and ubiquitination, FA core complex recruitment by FANCI was independent of FANCI DNA binding, ubiquitination and phosphorylation of the S/TQ cluster domain, and was also distinct from the ATR-mediated mechanism. All these data together show that FANCI has at least two independent functions within the FA pathway: (i) regulation of FANCD2 foci/ubiquitination and (ii) regulation of FA core complex foci.

Both FANCI phosphomutant (Ax6) and phosphomimetic mutant (Dx6), as well as the non-ubiquitinatable FANCI (K523R), significantly rescued MMC sensitivity in two different human FANCI-deficient cell lines. This data differs from studies in chicken DT40 cells, where the Ax6 mutant did not rescue sensitivity to ICL-inducing agents [24]. The discrepancy may be attributable to differences between human and chicken systems. It is particularly interesting that a phosphomutant FANCI (Ax6), while unable to support FANCD2 foci and ubiquitination, largely rescued MMC sensitivity. This data is consistent with an additional role of non-phosphorylated, non-ubiquitinated FANCI in the repair of ICLs. It is tempting to speculate that the two different pools of FANCI (phosphorylated-ubiquitinated and non-phosphorylated, non-ubiquitinated FANCI) may contribute independently to ICL-resistance by performing different functions. In support of this model, a function of unphosphorylated FANCI in the regulation of dormant origin firing was recently reported [49]. Our findings together with this report emphasize that both phosphorylated FANCI and unphosphorylated FANCI are needed for cellular resistance to ICLs. In the work presented by Chen and coworkers [49], FANCI phosphomimic mutant Dx6 cannot activate dormant origins or reverse MMC sensitivity in FANCI-depleted retinal pigment epithelial (RPE) cells. However, in our studies, the Dx6 mutant partially restored MMC resistance in FANCI-deficient patient-derived fibroblasts and FANCI-knockout HCT116 cells. This discrepancy may be explained by differential dependence on dormant origin firing for ICL tolerance among the cell lines.

Our results indicate that FA core complex binding to chromatin precedes, and is independent of, foci formation (S10D Fig). FANCI may affect the ability of the FA core complex to bind to chromatin in some cell lines such as U2OS. However, this phenomenon was not observed in HCT116 cells where FANCI was still required for the FA core complex to accumulate or persist at sites of DNA damage. Further investigations will be required to understand how this process occurs and to identify the reasons for these differences among cell lines.

ATR kinase activity was required for FA core complex foci formation. This was not unexpected, as ATR is involved in the activation of the FA pathway [23]. However, our findings suggest that ATR mediates FA core complex foci formation independently of FANCI phosphorylation, a well-characterized mechanism of ATR-mediated FA pathway activation [24]. Therefore, ATR will control the FA pathway at, at least, two steps. The relevant substrate of ATR in the FA core complex foci formation remains unknown. Possible candidates include FANCA, FANCG or FANCM, as they are phosphorylated by ATR [33, 50, 51].

The search for other factors with roles upstream of FA core complex foci formation uncovered two additional positive regulators: BRCA1 and USP1. This finding was surprising, since depletion of neither of these two proteins decrease the level of ubiquitination of FANCD2 or FANCI, and their ubiquitination is even increased in the case of USP1 depletion. These findings have, at least, two possible explanations: (i) residual levels of FA core complex foci formation observed in cells deficient for BRCA1 or USP1 are enough to induce normal (or increased) FANCD2-FANCI ubiquitination, or (ii) FA core complex foci formation and FANCD2-FANCI ubiquitination are uncoupled. Regardless of which of the two scenarios is correct, the fact that FA core complex foci and FANCD2-FANCI ubiquitination do not correlate suggests that the accumulation of the FA core complex at sites of DNA damage may serve additional functions. In support of this model, a mutation in FANCA (I939S) that does not impair FANCD2 monoubiquitination was recently described in an FA patient [52]. Also, the fact that depletion of USP1 results in increased ubiquitination of FANCD2-FANCI in the absence of DNA damage (our data and [53]) suggests that ubiquitination may not necessarily happen at sites of DNA damage, and may therefore be disconnected from FA core complex foci formation. It would be interesting to test if FA core complex foci are able to form in FANCA I939S and/or other separation-of-function mutants.

We showed that deubiquitination of FANCI by USP1 is an important step to recruit the FA core complex at sites of DNA damage. This result uncovers the existence of a feedback loop, centered on FANCI: not only ubiquitination, but also deubiquitination, is required for the proper functioning of the FA pathway. We have also, for the first time, uncovered a role of USP1 as a positive regulator of the FA pathway. More experiments are required to assess if deubiquitination of FANCI alone can explain ICL sensitivity of USP1-deficient cells.

It is important to note that BRCA1 or USP1 deficiency also leads to impaired FANCD2 foci formation [20, 54]. How BRCA1 promotes recruitment of both FANCD2 and FA core complex remains enigmatic. A recent *in vitro* study shows that BRCA1 acts in ICL repair upstream of DSB formation by facilitating eviction of the replicative helicase [55]. Therefore, this step could be required for FANCD2 and FA core complex foci formation

Taken together, we have optimized a protocol to visually detect the recruitment of the FA core complex to sites of DNA damage, which allowed us to identify regulators of FA core complex recruitment, and therefore, elucidate a previously unexplored aspect of the FA pathway. With the remarkable findings that FANCI, USP1 and BRCA1, as well as ATR and FANCM, promote FA core complex foci formation, we provide evidence that the FA pathway is a non-linear, tightly regulated pathway, with several proteins (ATR, FANCI, USP1 and BRCA1) performing roles at multiple stages of its activation (S13 Fig).

## Materials and Methods

### Cell lines and culture conditions

U2OS, HeLa, HCC1937 and TOV-21G were purchased from the American Type Culture Collections. FANCF-corrected TOV21G [56], SV40 transformed FA fibroblasts (326SV FA-G-/-, GM6914 FA-A-/-, PD20 FA-D2-/-) and their corrected counterparts [20, 57–59], hTERT-immortalized ATR deficient F02-98 fibroblasts and their corrected counterparts [23, 60], VU423 fibroblasts and VU423 corrected with chromosome 13[11], have been described. F010191 transformed fibroblasts [22] were a gift from Dr. Tony Huang (New York University). AG656 fibroblasts and AG656+FANCJ were a gift from Dr. Sharon Cantor (University of Massachusetts). EUFA1341 fibroblasts and EUFA1341+PALB2 were a gift from Dr. Paul Andreassen (Cincinnati Children’s Hospital). HCC1937 cells were cultured in RPMI supplemented with 15% FCS. HCT116 FANCI-/- were grown in McCoy’s 5A media supplemented with 10% FCS and 1% glutamine. All other cell lines were grown in DMEM supplemented with 10% FCS. All cells were maintained in a humidified 5% CO_2_ atmosphere at 37°C. Cells were treated with MMC (Sigma, M4287), cisplatin (Sigma, P4394), hydroxyurea (HU) (Sigma, H8627), AZD2281 (Selleckchem, S1060) ionizing radiation (IR) (JL Shepherd Mark I Cesium Irradiator (JL Shepherd & Associates)), ATR inhibitor VE-821 (Axon Medchem, 1893), ATM inhibitor KU55933 (Selleckchem, S1092) or USP1 inhibitor, ML323 [37](gift from Dr. Zhihao Zhuang, University of Delaware).

### Gene knockdown by RNA interference

siRNA transfections were conducted in 6-well plates using Lipofectamine RNAiMAX (Invitrogen), following the manufacturer’s instructions. 20nM siRNA was used in each transfection. siRNA sequences are provided in S1 Table.

### Generation of FANCI-null HCT116 cells

FANCI-null HCT116 cells were generated using recombinant adeno-associated virus (rAAV)-mediated gene targeting [61]. Conditional and knock-out rAAV vectors targeting FANCI exon 10 were constructed as described [62, 63]. The first round of targeting with the conditional vector replaced FANCI exon 10 with a floxed exon 10 along with a neomycin (Neo) drug selection cassette. G418-resistant clones were screened by PCR to confirm correct targeting and the Cre recombinase was subsequently used to remove the Neo selection cassette. Retention of the floxed exon 10 in the conditional allele was confirmed by PCR. The second round of gene targeting was performed with a knock-out vector that replaced exon 10 with a Neo-selection cassette. G418 resistant clones were again screened by PCR for correct targeting. Cre recombinase was subsequently used to remove both the Neo selection cassette and the floxed conditional allele and this resulted in viable FANCI-null clones. The PCR primers flanking FANCI exon 10 that were used to confirm both conditional and null alleles were FancIc_GG_LIF: GCAATGGCACAATCTTGG and FancIcond_GG_loxR: ATAGAACTTTCTGGCTTGCT.

### Cell fractionation and western blotting

Cell fractionations were prepared as described [17]. Briefly, cells were resuspended in buffer CSK (10mM PIPES, pH = 6.8, 100mM NaCl, 1mM EGTA, 1mM EDTA, 300mM Sucrose, 1.5mM MgCl2, 0.1% Triton-X-100 and protease inhibitors) and incubated in ice for 5min. Samples were centrifuged at 1500g for 5min. Supernatant was collected and stored (soluble fraction). Pellets (insoluble fraction) were washed once in CSK buffer and then resuspended in sample buffer (0.05 M Tris-HCl (pH 6.8), 2% SDS, 6% β-mercaptoethanol) and boiled for 5min. Western blotting was performed as described [64]. Briefly, cells were lysed in 0.05 M Tris-HCl (pH 6.8), 2% SDS, 6% β-mercaptoethanol and boiled for 5min. SDS-PAGE electrophoresis was done using NuPAGE 3% to 8% Tris-acetate or NuPAGE 4% to 12% Tris-glycine gels (Invitrogen) and proteins were transferred to a nitrocellulose membrane. Primary antibodies were diluted in blocking buffer (5% milk in TBS-Tween 20) and incubated overnight. Horseradish peroxidase—conjugated anti-mouse and anti-rabbit IgG (Amersham) were used as secondary antibodies. Images were acquired using ImageQuant LAS4000 system (GE Healthcare).

### Plasmids and cell transduction

Human FANCI coding sequence was amplified by PCR to include a Myc tag sequence at the 5’ end of the gene and cloned into pLentiX1-puro (a gift from Eric Campeau, Addgene plasmid # 20953) using SalI and XbaI sites. Ubiquitin C promoter was extracted from pUB-GFP (a gift from Connie Cepko, Addgene plasmid # 11155) using a SalI digestion. The resulting 1.2 Kb fragment was cloned into pLentiX1-MycFANCI at the SalI site. K523R, Ax2-Ax6, Dx6 and KKEE mutants of FANCI were generated using an overlap extension PCR method. Myc-USP1 and Myc-FANCG were cloned into pLentiX1-puro using the same strategy.

FANCI-containing lentiviruses were produced as described [65]. F010191 fibroblasts or FANCI-deficient HCT116 cells were transduced with fresh lentiviruses-containing supernatants and selected for 2 days with 2μg/ml puromycin. Fresh transductions were used in each experiment, as exogenous FANCI expression was lost when cells underwent prolonged culture after transduction.

### Immunofluorescence and microscopy

Cells were grown on coverslips and then fixed and permeabilized for 30 minutes using 4% paraformaldehyde (Santa Cruz, sc-281692) containing 0.5% Triton X-100. After fixation, cells were washed with PBS and then blocked for 15 minutes in PBS containing 3% BSA+ 0.1% Tween20. Primary antibodies were diluted in blocking buffer and incubation was performed at room temperature for one hour on a rocker. After 3 washes with PBS + 0.1% Tween20, cells were incubated with secondary antibodies diluted in blocking buffer for another 45 minutes. AlexaFluor 488 Goat Anti-Rabbit IgG and AlexaFluor 594 Goat Anti-Mouse IgG were used as secondary antibodies (Molecular Probes). At this point, 1μg/ml of 4',6-Diamidino-2-Phenylindole, Dihydrochloride (DAPI) was added to the cells and incubated for an additional 15 minutes. Cells were washed 3 times with PBS + 0.1% Tween20 and then coverslips were mounted using Vectashield Mounting Media (VectorLabs, H-1000). Image acquisitions were made with a TE2000 Nikon microscope equipped with a 60X immersion objective and a CCD camera (CoolSNAP ES, Photometrics). Images were acquired and analyzed using MetaVue (Universal Imaging) and ImageJ. Manual counting was used and cells containing more than 5 foci were scored as positive. When possible, cells were seeded in glass-bottom 96 well-plates (Greiner Bio-One, 655892) and images were acquired with Cellomics ArrayScan automated microscope equipped with a 20X objective. In this case, foci counting was automated using Cellomics software and reported as “foci per cell”.

### Laser-induced DNA damage

To generate localized DNA lesions, we used a published method [66] with some modifications. Cells were grown on 35mm glass bottom Fluorodish cell culture dishes (World Precision Instruments) for 24 hours before the experiment. One hour before the experiment, cells were placed in warm CO_2_-independent medium (Life Technologies). Cells were pre-sensitized with 10 μg/ml viable Hoechst dye 33258 (Sigma-Aldrich) for 5 min at 37°C. We performed laser microirradiation using an Nikon Ti fully-motorized inverted spinning disk confocal microscope (UltraView Vox, Perkin Elmer) equipped with a 37°C heating chamber and a 405 nm laser focused through a Nikon Plan Fluor 40x/1.3 NA oil objective. We set the laser output to 100% of maximum power to generate in three iterations in a restricted region detectable localized DNA damage that was restricted to the laser path, dependent on prior pre-sensitization of the cells, and without noticeable cytoxicity. Cells were fixed 30 minutes after generating DNA damage and stained with the indicated antibodies.

### Antibodies

The following primary antibodies were used: anti-FANCD2 (Abcam, ab2187), anti-FANCA (Bethyl, A301-980A), anti-Vinculin (Sigma, V9131), anti-ATR (Santa Cruz, sc-1887), anti-FANCI (Santa Cruz, sc-271316), anti-Actin (Santa Cruz, sc-1616), anti-BRCA1 (Santa Cruz, sc-6954), anti-CHK1 (Santa Cruz, sc-8408), anti-pCHK1 S345 (Cell Signalling, 23415), anti-FLAG M2 (Sigma, F1804), anti-γH2AX (Upstate, JBW3001), anti-CyclinA (Abcam, ab16726), anti-MYC tag 9E10 (Upstate, 05–419), anti-TRF1 (Abcam, ab10579), anti-RAD51 (CosmoBio, BAM-70-001-EX), anti-FANCJ (Sigma, B1312), anti-BRCA2 (Calbiochem, OP95), anti-ABRAXAS (Bethyl, A302-180A), anti-RAP80 (Bethyl, A300-763A). Anti-FANCG [67], FANCC [68], FANCE [69], FANCF [70] and FANCD2 pT691 [71] were gifts from Dr. Alan D’Andrea (Dana-Farber Cancer Institute, Boston). Anti-USP1 (C-ter) [38] was a gift from Dr. Tony Huang. Anti-PALB2 [72] was a gift from Drs. David Livingston and Bing Xia. Anti-FANCL and anti-FANCM were gifts from Dr. Weidong Wang.

### Cell synchronization and cell cycle analysis

Cell synchronization in M-phase was achieved by incubating cells in nocodazole (Sigma, M1404) at 0.1μg/ml for 16 hours. Floating cells (M-phase cells) were then recovered by shaking the culture flask, and re-seeded in 6 cm dishes and on coverslips in fresh medium without nocodazole. During a period of 21 hours, cells were collected every 3h for cell cycle analysis and immunostaining. For each time point, coverslips were irradiated with 10Gy IR 60min prior the collection. For cell cycle analysis, cells were fixed in ice-cold 70% ethanol for 16 hours. Then, ethanol was removed and cells were incubated in PBS + propidium iodide 40μg/ml + RNase A 0.1mg/ml for 30 minutes at 37°C. After that, cells were kept on ice until analysis by flow cytometry. Flow cytometry analyses were performed on BD FACSCanto II flow cytometers and analyzed with Flow Jo software.

### Survival assay

Cell survival was measured by a crystal violet absorbance-based assay. Cells were seeded onto 12-well plates at a density of 9000 cells/well. The next day, cells were treated with increasing concentrations of MMC and incubated for eight more days. After that, plates were processed as described [65].

### RT-PCR

mRNA was converted to cDNA using Superscript III Reverse Transcriptase (Invitrogen) following manufacturer’s instructions. To measure specific gene expression, the following sets of primers were used: GAPDH: 5’ GGAGTCAACGGATTTGGTCG 3’ and 5’ CTCCTGGAAGATGGTGATGG 3’; RNF8: 5’ AAGATGGGTGCGAGGTGACT 3’ and 5’ ACGCGCTCTGTTCAGCCAAA 3’.

### Statistics

All statistical analyses were done using Student’s t-test (2-tail). P value < 0.05 was considered significant.

## Supporting Information

S1 Table List of siRNA sequences. (DOCX)

S2 Table Summary of modulation of FA core complex foci formation by factors downstream of FANCD2-FANCI ubiquitination. (DOCX)

S1 Fig FA core complex forms foci at sites of DNA damage. (A) U2OS cells were left untreated or treated with 60ng/ml MMC, 2.5 μM cisplatin, 250μM HU or 10μM AZD2281 for 24h, or treated with 10Gy IR 8h before fixation. Then, cells were immunostained with anti-FANCA, FANCG, FANCE, FANCD2 or γH2AX antibodies. The percentage of cells with > 5 foci is shown (n = 3, mean ± SD). (B) HeLa cells were untreated or treated with 60ng/ml MMC for 24 hours, and immunostained with the indicated antibodies. (C) The percentage of cells with > 5 foci is shown for the experiments shown in (B). (n = 3, mean ± SD). (D) U2OS cells were treated with 60ng/ml MMC for 24 hours and immunostained with FANCA, FANCG and TRF1 antibodies. Representative images are shown. (E) GM6914 fibroblasts (FANCA-deficient) and complemented fibroblasts were treated with 60ng/ml MMC for 24h and immunostained with FANCA and γH2AX antibodies. Representative images are shown. (F) 326SV fibroblasts (FANCG-deficient) and complemented fibroblasts treated with 60ng/ml MMC for 24h and stained with FANCA and γH2AX antibodies. Representative images are shown. (G) Western blot analyses corresponding to the cell lines used in experiments shown in panels E and F.
(TIF)

S2 Fig FA core complex foci depend on the whole FA core complex and FANCM-FAAP24. (A) U2OS cells were transfected with indicated siRNAs, untreated or treated with MMC (60ng/ml) for 24h and immunostained with anti-FANCC or FANCL antibodies. Percentage of cells containing more than 5 foci is shown (n = 3, mean ± SD). (B) U2OS cells were transfected with indicated siRNAs, untreated or treated with MMC 60ng/ml MMC for 24h and immunostained with anti-FANCD2 antibody. Foci/cell were counted using automated software (n = 3, mean ± SD). (C) The same as panel B, but stained with a γH2AX antibody. (D) FANCF-deficient TOV21G cells and corrected cells were treated with MMC 60ng/ml MMC for 24h and then immunostained with the indicated antibodies. Representative images are shown. (E) Western blot analysis corresponding to the cell lines used in experiments shown in panel D. (F) U2OS cells were transfected with the indicated siRNAs, untreated or treated with MMC 60ng/ml MMC for 24h and immunostained with an anti-BRCA1 antibody. Foci/cell were counted using automated software (n = 3, mean ± SD). (G) mRNA levels detected by semiquantitative RT-PCR corresponding to the samples used in panel F.
(TIF)

S3 Fig FANCM depletion abrogates FA core complex foci formation without affecting FA core complex protein levels. (A) U2OS cells were transfected with siControl or siFANCM and treated with mitomycin C (MMC) 60ng/ml for 24h before fixation. Cells were immunostained with the indicated antibodies. (B) Western blot analyses corresponding to experiment shown in panel A. (C) U2OS cells transfected with siControl and siFANCC and immunoblotted with anti-FANCC antibody. (D) U2OS cells transfected with siControl and siFANCL and immunoblotted with anti-FANCL antibody.
(TIF)

S4 Fig Foci detection of exogenously expressed FANCG. (A) U2OS cells transduced with pMMP-FANCG or pLentiX1-mycFANCG were treated with MMC for 24h, and then fixed and stained with anti-FANCG or anti-MYCtag antibodies. (B) Cells from the experiment described in A were subjected to western blotting to assess FANCG expression level. (C) FANCG-deficient 326SV cells were transduced with the indicated constructs. Cells were plated at low density and treated with increasing concentrations of MMC. The cell-surviving fraction after 6 days, compared to untreated cells is shown.
(TIF)

S5 Fig FA core complex foci form in S-G2 phases of the cell cycle. (A) U2OS cells were untreated or treated with 10 Gy IR and fixed at the indicated time points. The percentage of cells with >5 foci is shown. (B) Cells were transfected with indicated siRNAs and treated with 10 Gy IR 2 hours before fixation. Then, cells were immunostained with FANCD2 and cyclin A antibodies. The graph shows the percentage of cyclin A-positive and -negative cells in the FANCD2-foci containing cells (n = 3, mean ± SD). (C) Same conditions as in panel B, immunostained with BRCA1 and Cyclin A antibodies. The percentage of cells with > 5 foci is shown (n = 3, mean ± SD). (D) Western blot analyses corresponding to the samples used in experiments shown in panels B and C.
(TIF)

S6 Fig ATR, but not ATM, is required for FA core complex foci formation. (A) U2OS cells were transfected with the indicated siRNAs, untreated or treated with MMC 60ng/ml for 24h and immunostained with anti-FANCA or FANCG antibodies. Foci/cell were counted using automated software (n = 3, mean ± SD). (B) U2OS cells were transfected with the indicated siRNAs, untreated or treated with MMC 60ng/ml for 24h and immunostained with anti-FANCC or FANCL antibodies. Percentage of cells containing 5 or more foci were counted (n = 3, mean ± SD). (C) U2OS cells were pre-treated with an ATM inhibitor (KU55933) (10 μM) for 2 hours, then left untreated or treated with MMC 60ng/ml for another 24 hours before fixation and immunostaining. The percentage of cells with > 5 foci is shown (n = 3, mean ± SD). (D) Western blotting analyses using an anti-phosphoFANCD2 T691 antibody. Cells were pre-treated with the ATM inhibitor or DMSO for 2h, then irradiated with 10 Gy. Protein samples were prepared 8 hours later. (*) indicates non-specific band.
(TIF)

S7 Fig FANCI, but not FANCD2, is required for FA core complex foci formation. (A) U2OS cells were transfected with the indicated siRNAs, treated with MMC (60 ng/ml) for 24 hours and then fixed and stained with anti-FANCC and anti-FANCL antibodies. Cells with >5 foci were counted and the percentage of positive cells is shown (n = 3, mean ± SD). (B) FANCI-deficient F010191 cells were transduced with the indicated constructs, treated with MMC and then fixed and stained with anti-FANCA and anti-FANCD2. Cells with >5 foci were counted and the percentage of positive cells is shown (n = 3, mean ± SD). (C) Representative images corresponding to experiment described in B. (D) Protein samples corresponding to experiment described in B, were subjected to western-blotting.
(TIF)

S8 Fig Effect of FANCI ubiquitination and phosphorylation site mutations on FANCD2-FANCI ubiquitination. Western blotting analyses of FANCD2 and myc-FANCI. FANCI-deficient F010191 cells were transduced with myc-FANCI wild-type or mutant forms, untreated or treated with MMC 60ng/ml for 24h before collecting protein samples.
(TIF)

S9 Fig Effect of FANCI ubiquitination and phosphorylation site mutations on foci formation, MMC sensitivity and FANCD2-FANCI ubiquitination in FANCI-deficient HCT116 cells. (A) FANCI-deficient HCT116 cells were transduced with wild-type or mutant forms of FANCI. The cells were treated with MMC 60ng/ml for 24h and then fixed and immunostained with the indicated antibodies. The percentage of foci-positive cells is shown (n = 3, mean ± SD). (B) Representative images corresponding to the experiment described in panel A. (C) Western blotting analyses of FANCD2 and myc-FANCI. FANCI-deficient HCT116 cells were transduced with myc-FANCI wild-type or mutant forms, untreated or treated with MMC 60ng/ml for 24h before collecting protein samples. (D) FANCI-deficient HCT116 cells were transduced with wild-type and mutant forms of FANCI, plated at low density and treated with increasing concentrations of MMC. The cell-surviving fraction after 5 days, compared to untreated cells is shown (n = 3, mean ± SEM).
(TIF)

S10 Fig Regulation of FANCA binding to chromatin by FANCI and ATR. (A) U2OS cells were transfected with the indicated siRNAs and treated with MMC 60ng/ml for 24h. The number of foci/cell (n = 3, mean ± SD) is shown. (*) Indicates p < 0.05. (B) Immunoblot analyses corresponding to subcellular fractions of U2OS cells transfected with the indicated siRNAs and treated with MMC for 24 hours. (S): soluble fraction; (I): insoluble fraction. Ratio of insoluble/soluble FANCA, relative to control cells is provided. The data corresponds to 4 independent experiments (mean ± SD). (C) Same as panel B. (D) Immunoblot analyses corresponding to subcellular fractions of U2OS cells untreated or treated with MMC 60ng/ml for 24h. (S): soluble fraction; (I): insoluble fraction. (E) Immunoblot analyses corresponding to subcellular fractions of HCT116 untransfected or transfected with the indicated siRNAs and treated with MMC 60ng/ml for 24h.
(TIF)

S11 Fig BRCA1 promotes FA core complex foci formation, while FANCJ, BRCA2, PALB2, CtIP, ABRAXAS or RAP80 is not required for the FA core complex foci formation. (A) U2OS cells were transfected with the indicated siRNAs, treated with MMC 60ng/ml for 24h., then fixed and immunostained with anti-FANCC and anti-FANCL antibodies. The percentage of cells with > 5 foci (n = 3, mean ± SD) is shown. (B) HeLa cells were transfected with the indicated siRNAs and treated with MMC 60ng/ml for 24h. The percentage of cells with > 5 foci (n = 3, mean ± SD) is shown. (C) Immunoblot analyses corresponding to the experiment described in panel A. (D) FANCJ-deficient AG656 and complemented fibroblasts treated with MMC 60ng/ml for 24 hours before fixation and staining. The percentage of cells with > 5 foci is shown in upper panel. Lower panel corresponds to western blotting confirming FANCJ status in these cell lines. (E) BRCA2-deficient VU423T and complemented fibroblasts were treated with MMC 60ng/ml for 24 hours before fixation and staining. Percentage of cells with > 5 foci is shown in upper panel. Lower panel corresponds to western blotting confirming BRCA2 status in these cell lines. (F) PALB2-deficient EUFA1341 and complemented fibroblasts were treated with MMC 60ng/ml for 24 hours before fixation and staining. Percentage of cells with > 5 foci is shown in upper panel. Lower panel corresponds to western blotting confirming PALB2 status in these cell lines. (G) and (H) U2OS cells were transfected with the indicated siRNAs and treated with MMC 60ng/ml for 24h. The number of foci/cell (n = 3, mean ± SD) is shown in left panels. Western blot confirming protein knock-down is shown in right panels.
(TIF)

S12 Fig USP1 catalytic activity is required for FA core complex foci formation. (A) U2OS cells were transfected with the indicated siRNAs, treated with IR 10 Gy, fixed 8 hours later, and immunostained with anti-FANCC and anti-FANCL antibodies. Percentage of cells with 5 or more foci is shown (n = 3, mean ± SD). (B) Immunoblot analyses corresponding to U2OS cells treated with the indicated doses of ML323 for 10 hours. (C) U2OS cells were transfected with the indicated combinations of siRNA and plasmid 48 hours before treatment (IR 10 Gy, fixed 8 hours later). The percentage of cells containing > 5 FANCD2 foci (n = 3, mean ± SD) is shown. In samples transfected with myc-tagged USP1 wild-type or myc-tagged USP1 C90S, only USP1-expressing cells (myc-positive) were included in the analysis. Immunoblot analyses corresponding to experiment shown in Fig 7C and 7D and S11C Fig.
(TIF)

S13 Fig Proposed model for the regulation of the FA pathway. FANCI, BRCA1, USP1 and ATR positively regulate FA core complex foci formation. Non-ubiquitinated/non-phosphorylated FANCI is able to perform this function, independently of FANCD2. USP1 promotes FA core complex foci by deubiquitinating FANCI. ATR kinase activity is required. Candidate substrates include FANCM, FANCA or other members of the FA core complex. At the site of DNA damage, the FA core complex may help promote recruitment of ubiquitinated FANCD2-FANCI.
(TIF)
