# Supplementary material for: FANCI Regulates Recruitment of the FA Core Complex at Sites of DNA Damage Independently of FANCD2
Source: PLoS Genet. 2015 Oct 2;11(10):e1005563. doi: 10.1371/journal.pgen.1005563 (PMC4592014; doi:10.1371/journal.pgen.1005563)
Supplement: S1 Table — (DOCX) [file pgen.1005563.s001.docx]

**S1 Table. List of siRNA sequences**

| Gene | siRNA ID | Sequence (5’-3’) |
| --- | --- | --- |
| FANCA | siFANCA | AAGGGUCAAGAGGGAAAAAUA |
| FANCC | siFANCC | GUGAGAGAAAUUGUCUGAGAA, |
| FANCF | siFANCF | CUUAUUAGCUCUUCGUAGU |
| FANCL | siFANCL | GACAAGAGCUGUAUGCACU |
| FANCM | siFANCM | aagcucauaaagcucucggaa |
| FAAP24 | siFAAP24_1 | CCGGAUGAGUGAACAAUACUU |
|  | siFAAP24_2 | AUUUUCGAGGAUGGCUUGACA |
| RNF8 | siRNF8 | AGAAUGAGCUCCAAUGUAUUU |
| ATR | siATR | AACCUCCGUGAUGUUGCUUGA |
| FANCD2 | siFANCD2_1 | AACAGCCAUGGAUACACUUGA |
|  | siFANCD2_2 | CUGGCUCAGGAUUCUAAUGUA |
| FANCI | siFANCI_1 | CUGGCUAAUCACCAAGCUUAA |
|  | siFANCI_2 | UUGAAUUUACUUAGCAGUCAA |
|  | siFANCI_3 | CACGGGCAUCUGGGAGAUAUA |
| BRCA1 | siBRCA1_1 | AAGGAACCUGUCUCCACAAAG |
|  | siBRCA1_2 | GGAACCUGUCUCCACAAAG |
| USP1 | siUSP1_1 | UCGGCAAUACUUGCUAUCUUA |
|  | siUSP1_2 | CGACAGCUAUGGAUUAUUU |
|  | siUSP1_3 | GAGAAGGACUUUCUGAAUU |
| ABRAXAS | siABRAXAS | CGUUUAGAGAGAGGCUGCUUCACAA |
| RAP80 | siRAP80 | UUUAAUUGAGCUUUCCUGGAA |
| CtIP | siCtIP_1 | AAGCUAAAACAGGAACGAAUC |
|  | siCtIP_2 | AAUCCACAACAUAAUCCUAAU |
| 53BP1 | si53BP1 | GAGAGCAGAUGAUCCUUUA |
| RIF1 | siRIF1 | GCAUUGACUUCUCACCAU A |
