# Supplementary material for: FANCI Regulates Recruitment of the FA Core Complex at Sites of DNA Damage Independently of FANCD2
Source: PLoS Genet. 2015 Oct 2;11(10):e1005563. doi: 10.1371/journal.pgen.1005563 (PMC4592014; doi:10.1371/journal.pgen.1005563)
Supplement: S2 Table — (DOCX) [file pgen.1005563.s002.docx]

**S2 Table. Summary of modulation of FA core complex foci formation by factors downstream of FANCD2-FANCI ubiquitination**

| Gene | Method | Cell line | FANCA foci (Deficient compared to control cell line) |
| --- | --- | --- | --- |
| BRCA1 | siRNA | U2OS | Reduced |
|  | Deficient/complemented epithelial cells | HCC1937 | Reduced |
| BRCA2 | Deficient/complemented fibroblasts | VU423T | Normal |
| CtIP | siRNA | U2OS | Normal |
| FANCJ/BRIP1 | Deficient/complemented fibroblasts | AG656 | Normal |
| FANCN/PALB2 | Deficient/complemented fibroblasts | EUFA1341 | Normal |
| FANCP/SLX4 | siRNA | U2OS | Normal |
| FANCQ/XPF | siRNA | U2OS | Normal |
| USP1 | siRNA | U2OS | Reduced |
|  | Chemical inhibitor (ML323) | U2OS | Reduced |
