# Supplementary figures and images for: FANCI Regulates Recruitment of the FA Core Complex at Sites of DNA Damage Independently of FANCD2
Source: PLoS Genet. 2015 Oct 2;11(10):e1005563. doi: 10.1371/journal.pgen.1005563 (PMC4592014; doi:10.1371/journal.pgen.1005563)

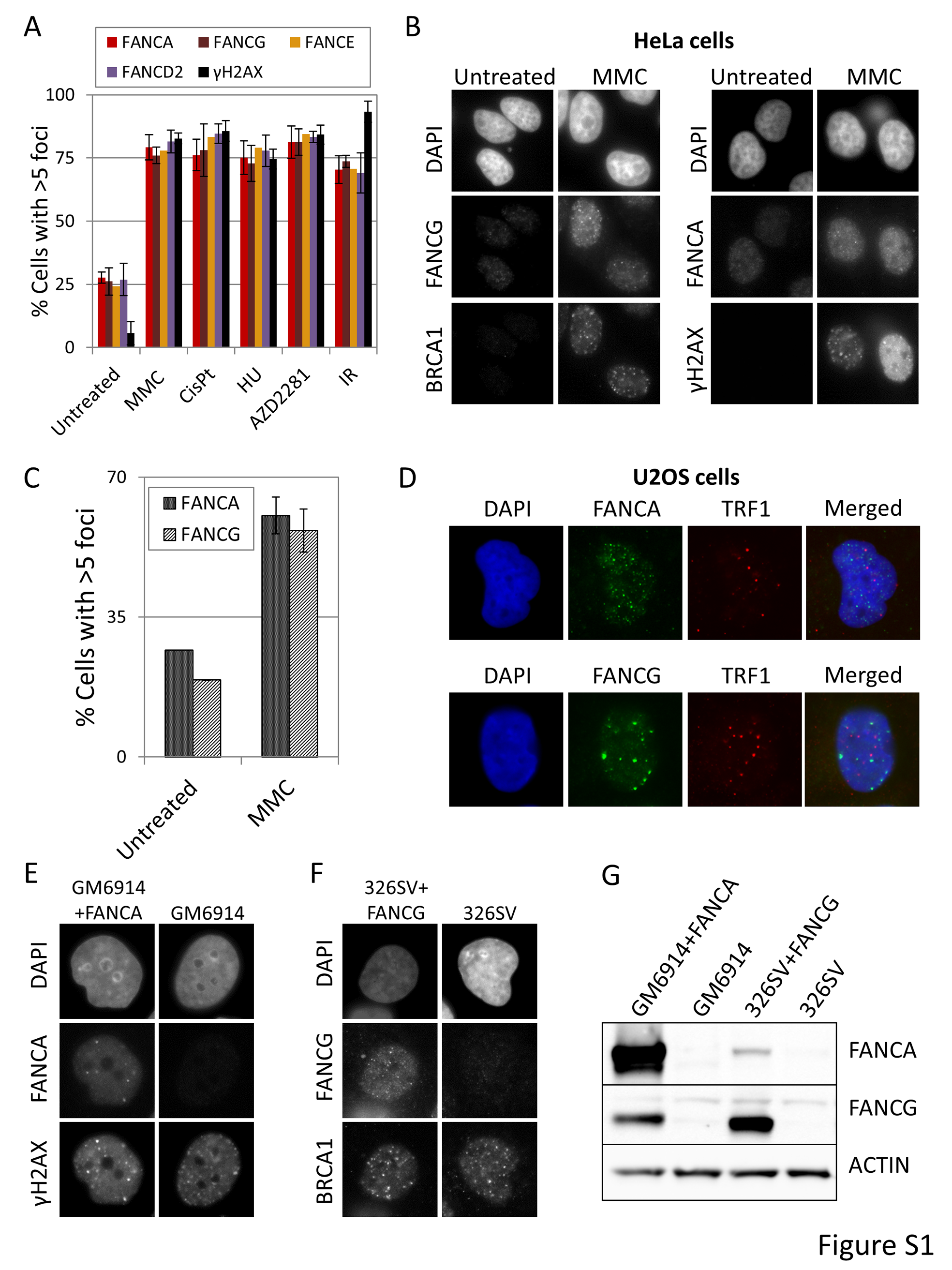

Supplement: S1 Fig — (A) U2OS cells were left untreated or treated with 60ng/ml MMC, 2.5 μM cisplatin, 250μM HU or 10μM AZD2281 for 24h, or treated with 10Gy IR 8h before fixation. Then, cells were immunostained with anti-FANCA, FANCG, FANCE, FANCD2 or γH2AX antibodies. The percentage of cells with > 5 foci is shown (n = 3, mean ± SD). (B) HeLa cells were untreated or treated with 60ng/ml MMC for 24 hours, and immunostained with the indicated antibodies. (C) The percentage of cells with > 5 foci is shown for the experiments shown in (B). (n = 3, mean ± SD). (D) U2OS cells were treated with 60ng/ml MMC for 24 hours and immunostained with FANCA, FANCG and TRF1 antibodies. Representative images are shown. (E) GM6914 fibroblasts (FANCA-deficient) and complemented fibroblasts were treated with 60ng/ml MMC for 24h and immunostained with FANCA and γH2AX antibodies. Representative images are shown. (F) 326SV fibroblasts (FANCG-deficient) and complemented fibroblasts treated with 60ng/ml MMC for 24h and stained with FANCA and γH2AX antibodies. Representative images are shown. (G) Western blot analyses corresponding to the cell lines used in experiments shown in panels E and F. (TIF) [file pgen.1005563.s003.tif]

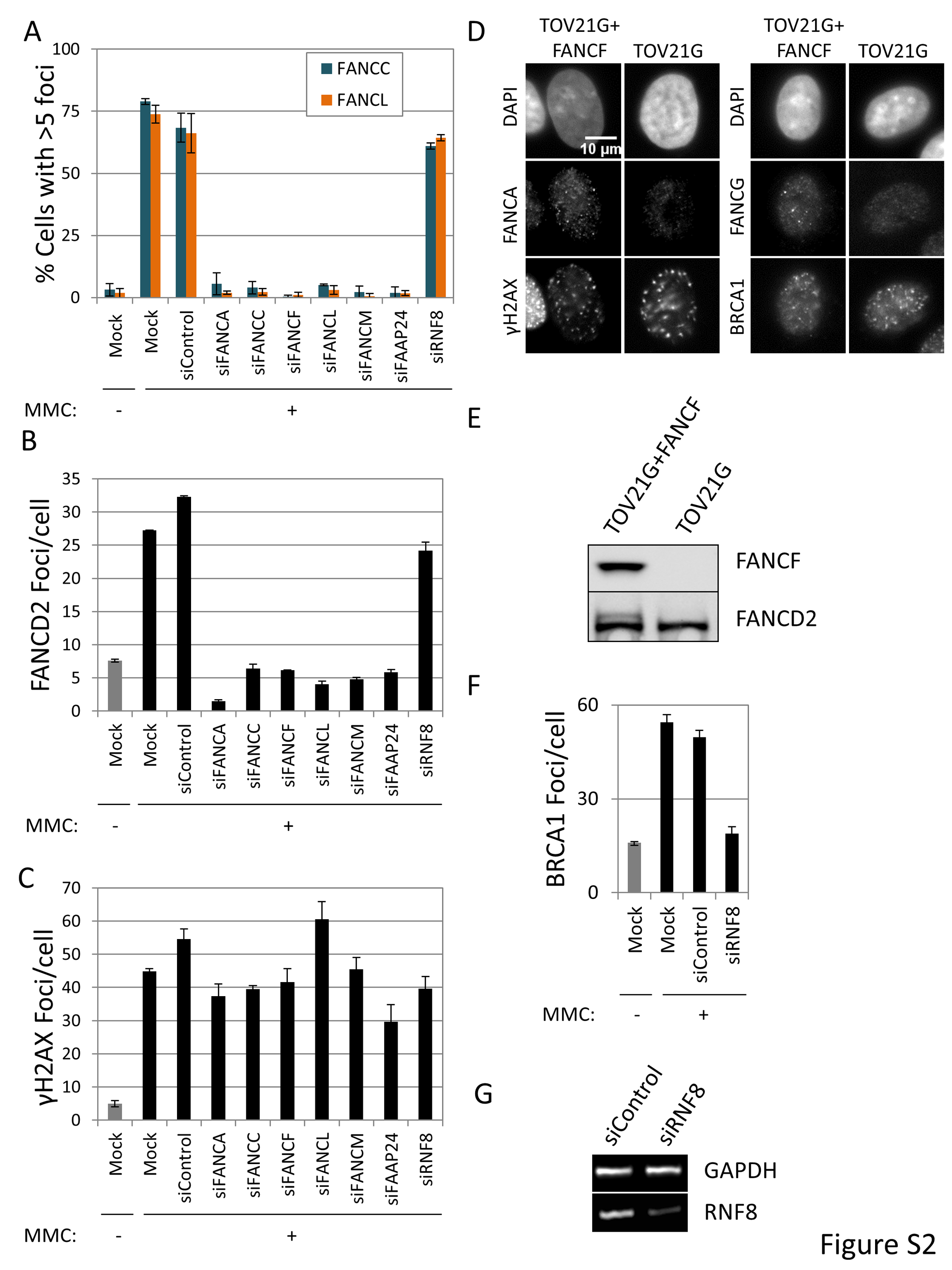

Supplement: S2 Fig — (A) U2OS cells were transfected with indicated siRNAs, untreated or treated with MMC (60ng/ml) for 24h and immunostained with anti-FANCC or FANCL antibodies. Percentage of cells containing more than 5 foci is shown (n = 3, mean ± SD). (B) U2OS cells were transfected with indicated siRNAs, untreated or treated with MMC 60ng/ml MMC for 24h and immunostained with anti-FANCD2 antibody. Foci/cell were counted using automated software (n = 3, mean ± SD). (C) The same as panel B, but stained with a γH2AX antibody. (D) FANCF-deficient TOV21G cells and corrected cells were treated with MMC 60ng/ml MMC for 24h and then immunostained with the indicated antibodies. Representative images are shown. (E) Western blot analysis corresponding to the cell lines used in experiments shown in panel D. (F) U2OS cells were transfected with the indicated siRNAs, untreated or treated with MMC 60ng/ml MMC for 24h and immunostained with an anti-BRCA1 antibody. Foci/cell were counted using automated software (n = 3, mean ± SD). (G) mRNA levels detected by semiquantitative RT-PCR corresponding to the samples used in panel F. (TIF) [file pgen.1005563.s004.tif]

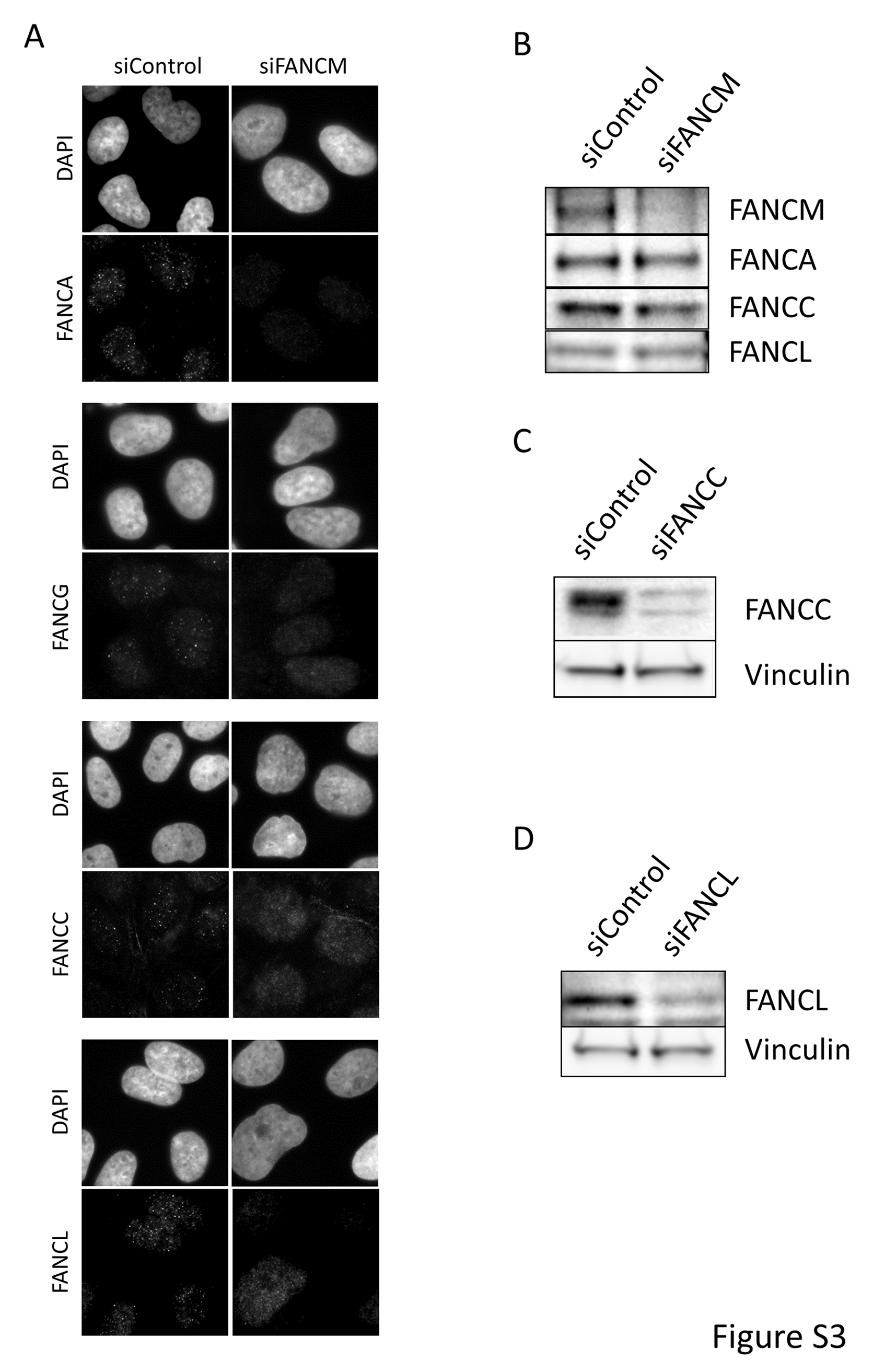

Supplement: S3 Fig — (A) U2OS cells were transfected with siControl or siFANCM and treated with mitomycin C (MMC) 60ng/ml for 24h before fixation. Cells were immunostained with the indicated antibodies. (B) Western blot analyses corresponding to experiment shown in panel A. (C) U2OS cells transfected with siControl and siFANCC and immunoblotted with anti-FANCC antibody. (D) U2OS cells transfected with siControl and siFANCL and immunoblotted with anti-FANCL antibody. (TIF) [file pgen.1005563.s005.tif]

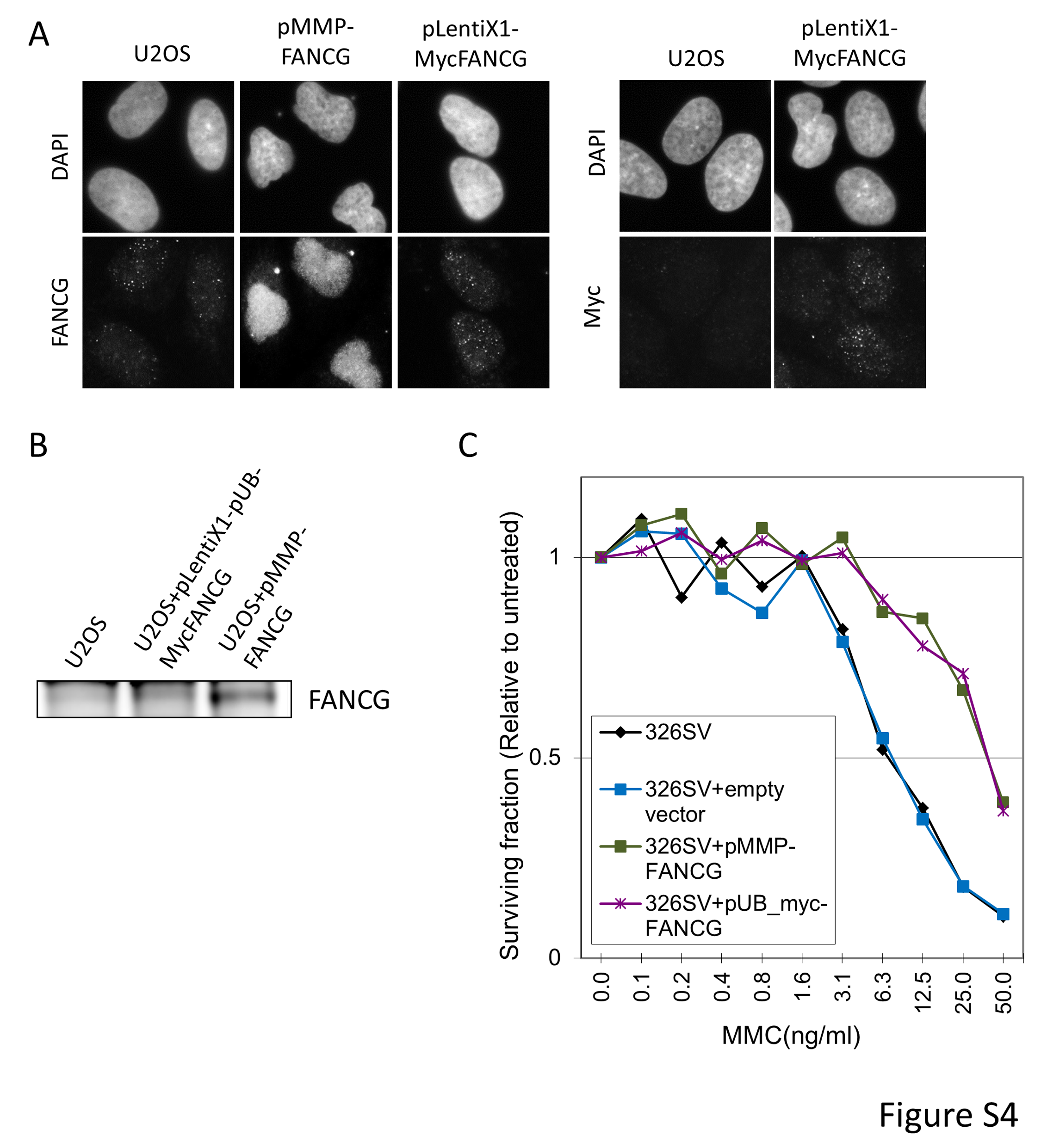

Supplement: S4 Fig — (A) U2OS cells transduced with pMMP-FANCG or pLentiX1-mycFANCG were treated with MMC for 24h, and then fixed and stained with anti-FANCG or anti-MYCtag antibodies. (B) Cells from the experiment described in A were subjected to western blotting to assess FANCG expression level. (C) FANCG-deficient 326SV cells were transduced with the indicated constructs. Cells were plated at low density and treated with increasing concentrations of MMC. The cell-surviving fraction after 6 days, compared to untreated cells is shown. (TIF) [file pgen.1005563.s006.tif]

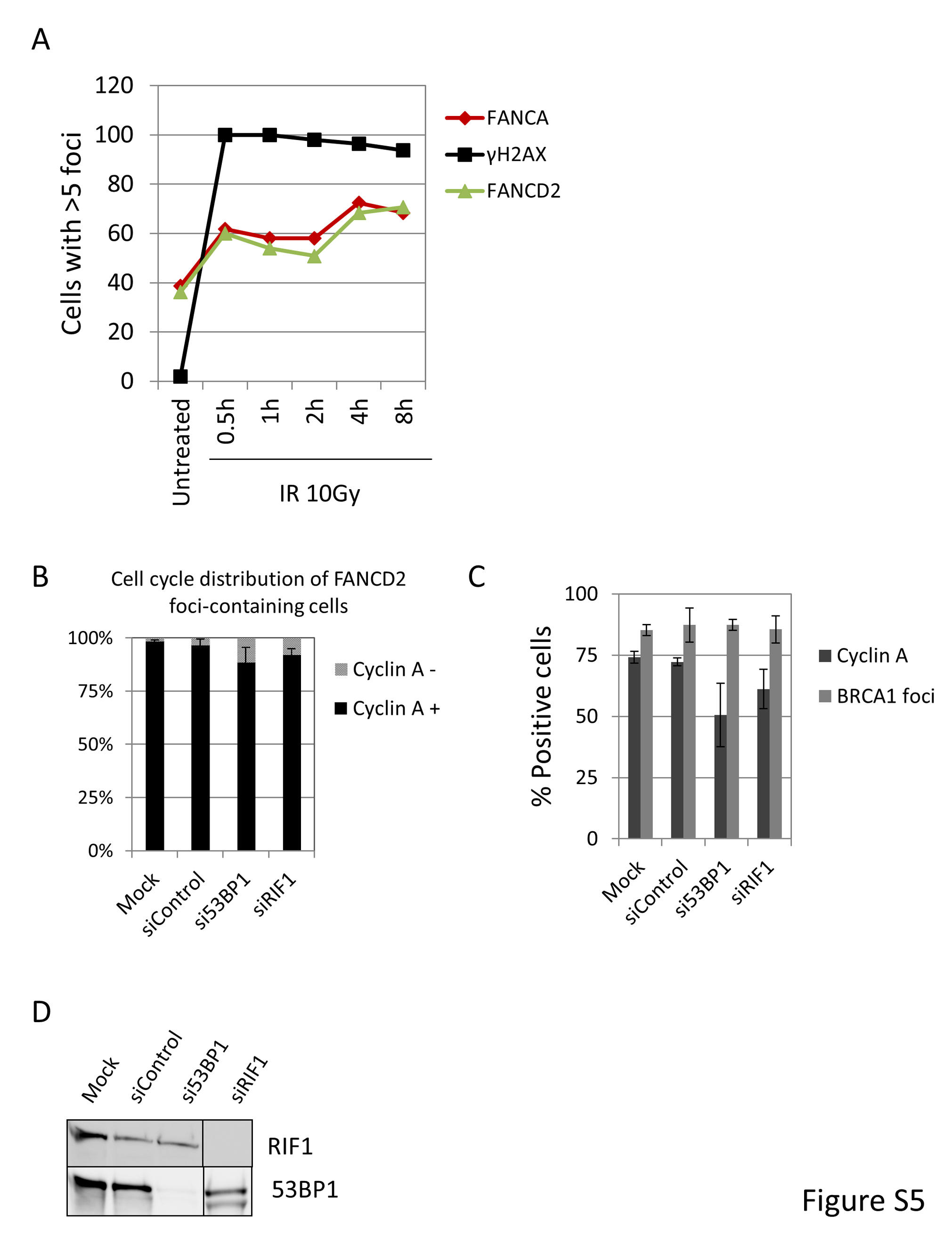

Supplement: S5 Fig — (A) U2OS cells were untreated or treated with 10 Gy IR and fixed at the indicated time points. The percentage of cells with >5 foci is shown. (B) Cells were transfected with indicated siRNAs and treated with 10 Gy IR 2 hours before fixation. Then, cells were immunostained with FANCD2 and cyclin A antibodies. The graph shows the percentage of cyclin A-positive and -negative cells in the FANCD2-foci containing cells (n = 3, mean ± SD). (C) Same conditions as in panel B, immunostained with BRCA1 and Cyclin A antibodies. The percentage of cells with > 5 foci is shown (n = 3, mean ± SD). (D) Western blot analyses corresponding to the samples used in experiments shown in panels B and C. (TIF) [file pgen.1005563.s007.tif]

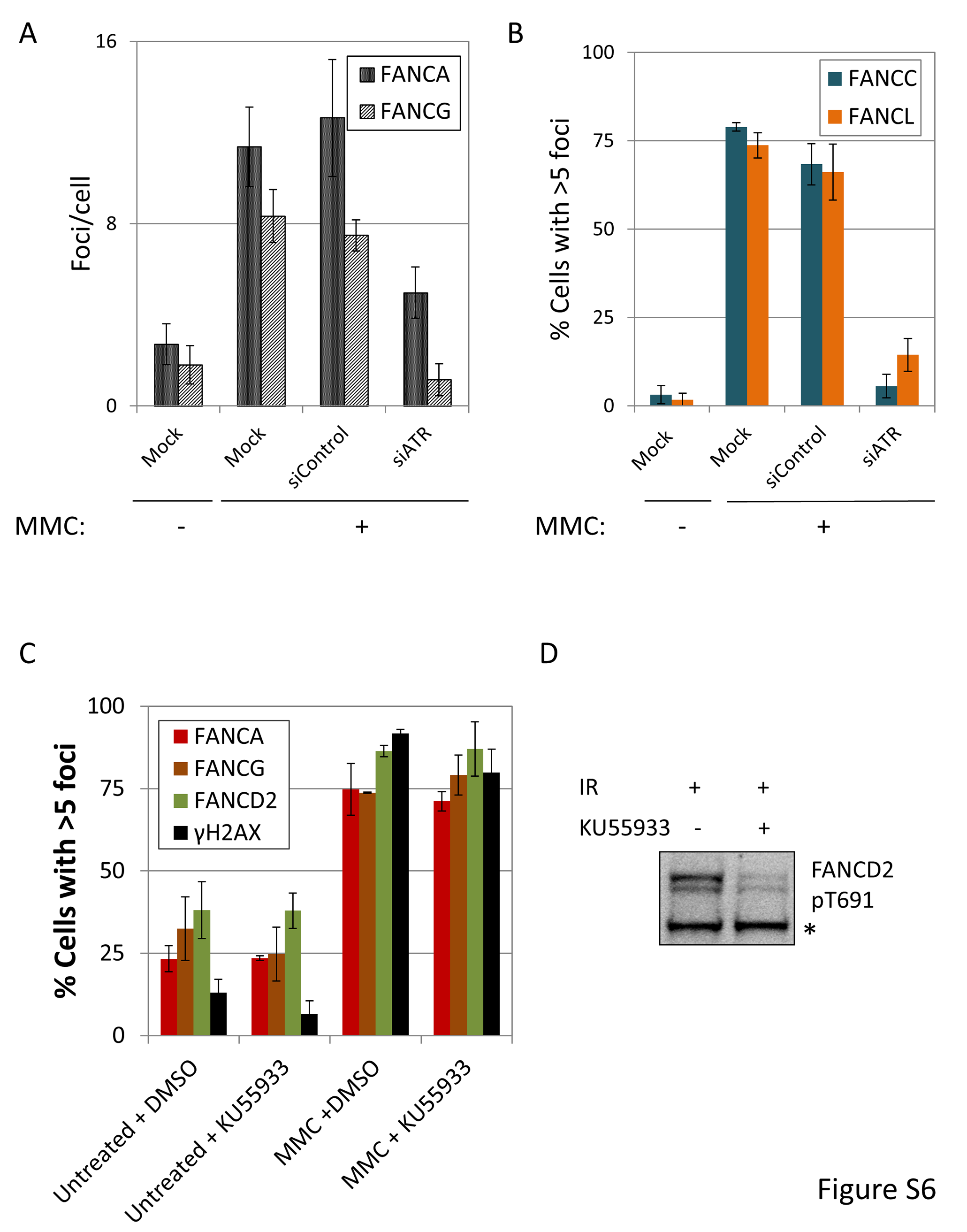

Supplement: S6 Fig — (A) U2OS cells were transfected with the indicated siRNAs, untreated or treated with MMC 60ng/ml for 24h and immunostained with anti-FANCA or FANCG antibodies. Foci/cell were counted using automated software (n = 3, mean ± SD). (B) U2OS cells were transfected with the indicated siRNAs, untreated or treated with MMC 60ng/ml for 24h and immunostained with anti-FANCC or FANCL antibodies. Percentage of cells containing 5 or more foci were counted (n = 3, mean ± SD). (C) U2OS cells were pre-treated with an ATM inhibitor (KU55933) (10 μM) for 2 hours, then left untreated or treated with MMC 60ng/ml for another 24 hours before fixation and immunostaining. The percentage of cells with > 5 foci is shown (n = 3, mean ± SD). (D) Western blotting analyses using an anti-phosphoFANCD2 T691 antibody. Cells were pre-treated with the ATM inhibitor or DMSO for 2h, then irradiated with 10 Gy. Protein samples were prepared 8 hours later. (*) indicates non-specific band. (TIF) [file pgen.1005563.s008.tif]

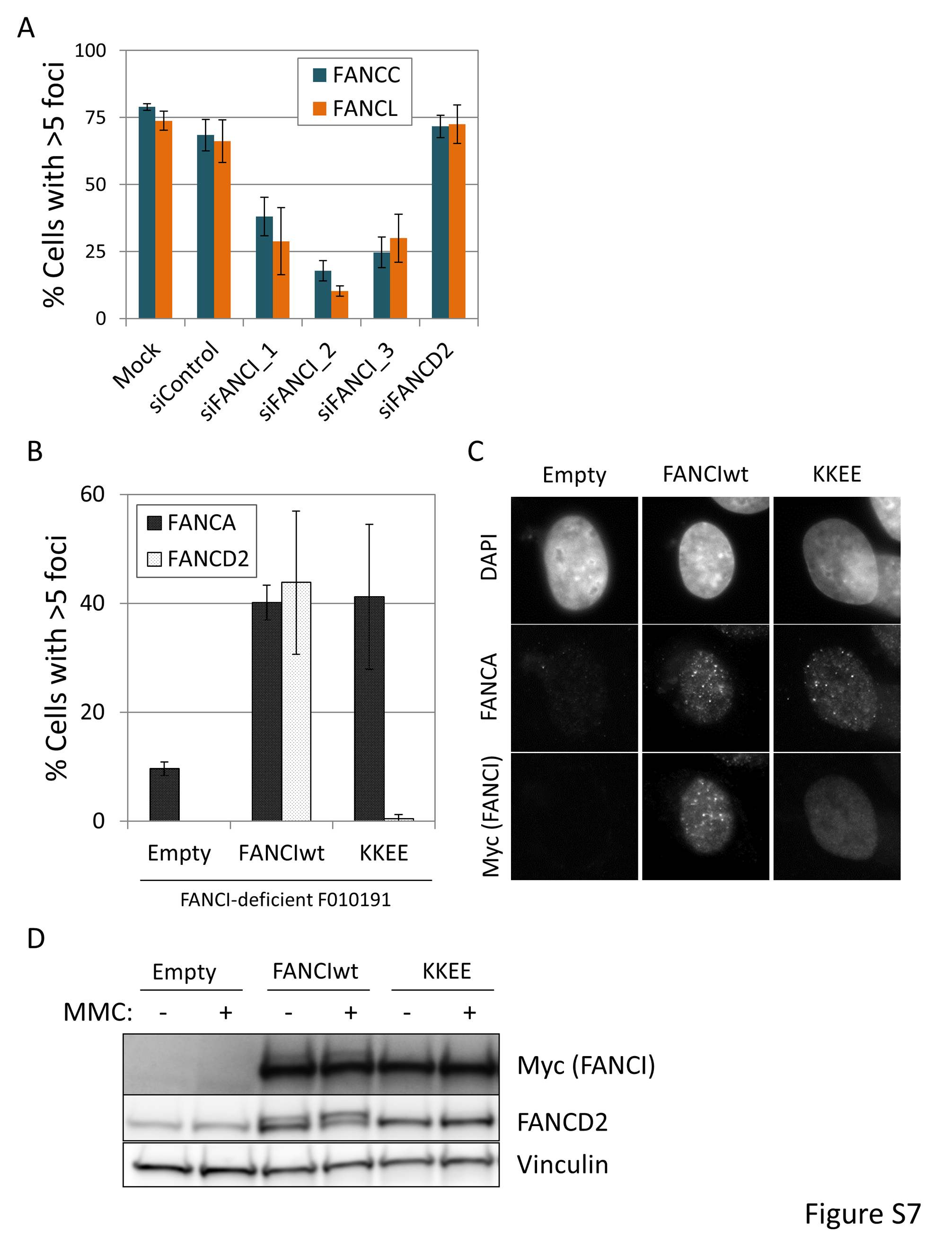

Supplement: S7 Fig — (A) U2OS cells were transfected with the indicated siRNAs, treated with MMC (60 ng/ml) for 24 hours and then fixed and stained with anti-FANCC and anti-FANCL antibodies. Cells with >5 foci were counted and the percentage of positive cells is shown (n = 3, mean ± SD). (B) FANCI-deficient F010191 cells were transduced with the indicated constructs, treated with MMC and then fixed and stained with anti-FANCA and anti-FANCD2. Cells with >5 foci were counted and the percentage of positive cells is shown (n = 3, mean ± SD). (C) Representative images corresponding to experiment described in B. (D) Protein samples corresponding to experiment described in B, were subjected to western-blotting. (TIF) [file pgen.1005563.s009.tif]

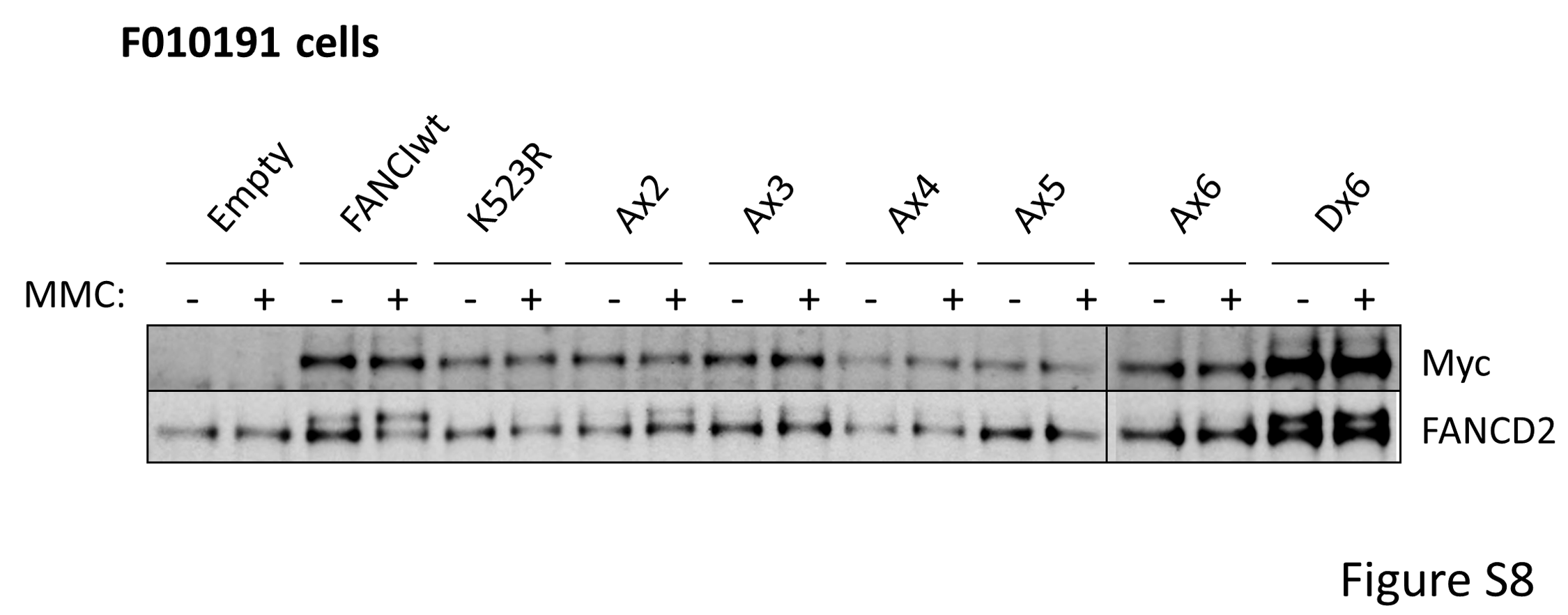

Supplement: S8 Fig — Western blotting analyses of FANCD2 and myc-FANCI. FANCI-deficient F010191 cells were transduced with myc-FANCI wild-type or mutant forms, untreated or treated with MMC 60ng/ml for 24h before collecting protein samples. (TIF) [file pgen.1005563.s010.tif]

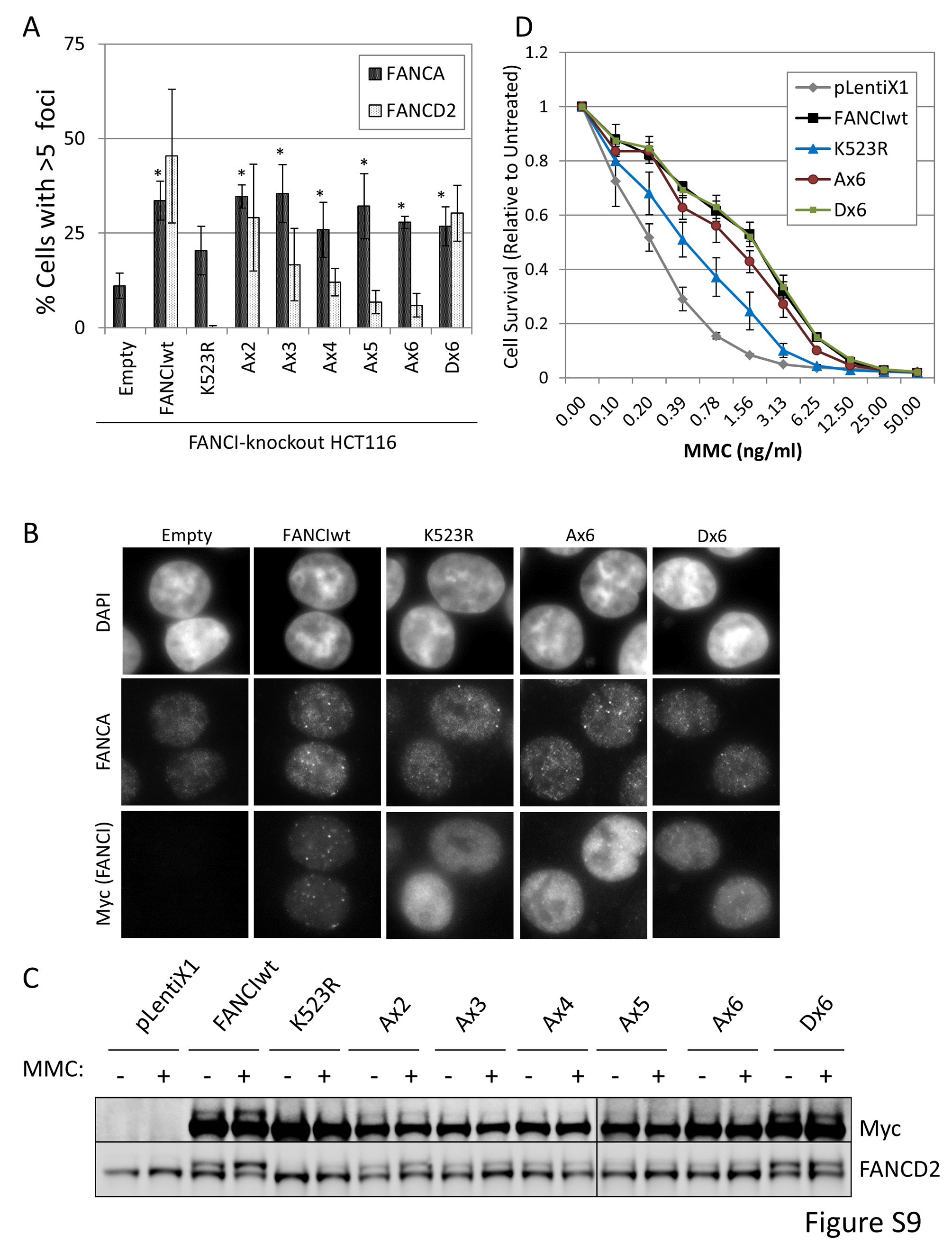

Supplement: S9 Fig — (A) FANCI-deficient HCT116 cells were transduced with wild-type or mutant forms of FANCI. The cells were treated with MMC 60ng/ml for 24h and then fixed and immunostained with the indicated antibodies. The percentage of foci-positive cells is shown (n = 3, mean ± SD). (B) Representative images corresponding to the experiment described in panel A. (C) Western blotting analyses of FANCD2 and myc-FANCI. FANCI-deficient HCT116 cells were transduced with myc-FANCI wild-type or mutant forms, untreated or treated with MMC 60ng/ml for 24h before collecting protein samples. (D) FANCI-deficient HCT116 cells were transduced with wild-type and mutant forms of FANCI, plated at low density and treated with increasing concentrations of MMC. The cell-surviving fraction after 5 days, compared to untreated cells is shown (n = 3, mean ± SEM). (TIF) [file pgen.1005563.s011.tif]

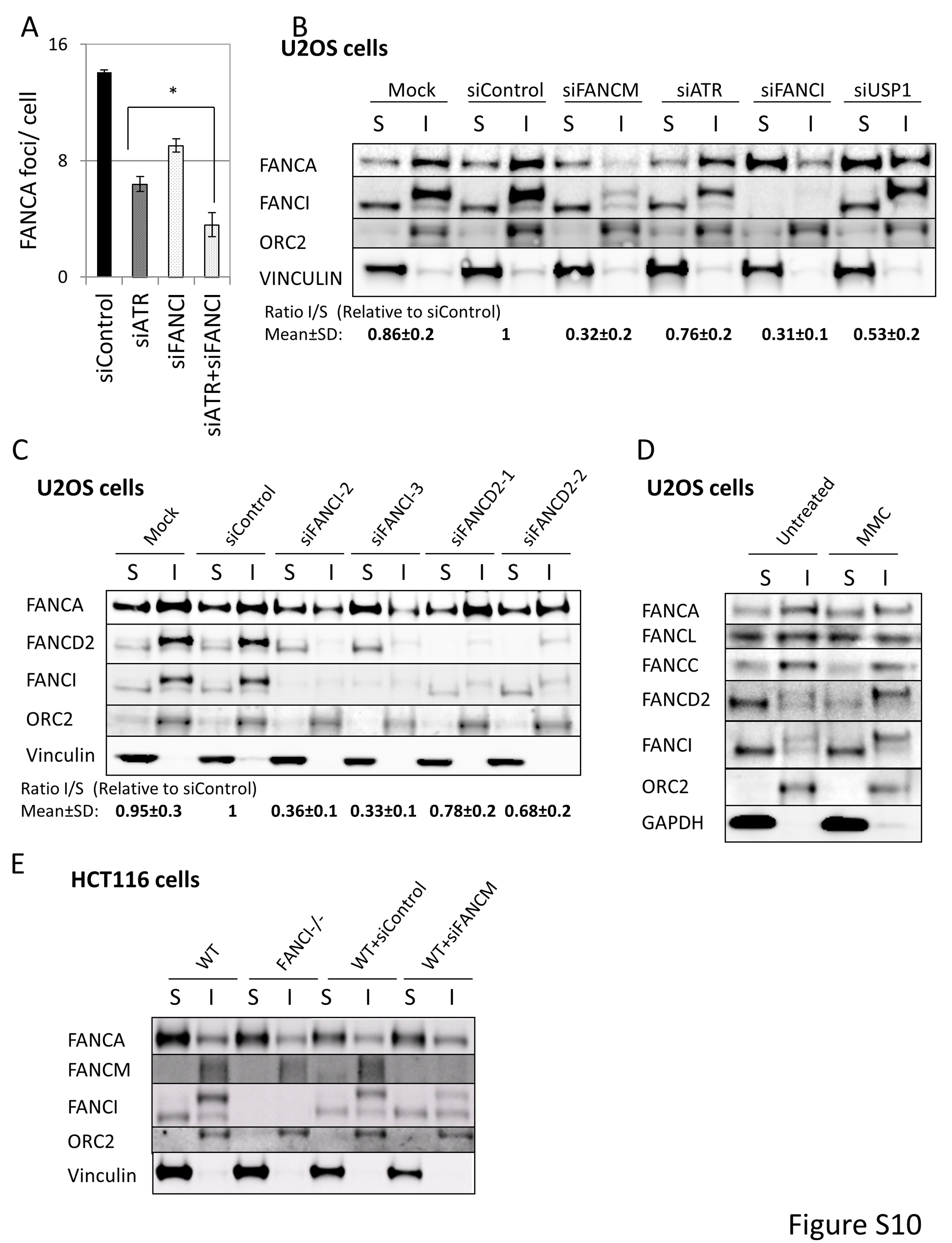

Supplement: S10 Fig — (A) U2OS cells were transfected with the indicated siRNAs and treated with MMC 60ng/ml for 24h. The number of foci/cell (n = 3, mean ± SD) is shown. (*) Indicates p < 0.05. (B) Immunoblot analyses corresponding to subcellular fractions of U2OS cells transfected with the indicated siRNAs and treated with MMC for 24 hours. (S): soluble fraction; (I): insoluble fraction. Ratio of insoluble/soluble FANCA, relative to control cells is provided. The data corresponds to 4 independent experiments (mean ± SD). (C) Same as panel B. (D) Immunoblot analyses corresponding to subcellular fractions of U2OS cells untreated or treated with MMC 60ng/ml for 24h. (S): soluble fraction; (I): insoluble fraction. (E) Immunoblot analyses corresponding to subcellular fractions of HCT116 untransfected or transfected with the indicated siRNAs and treated with MMC 60ng/ml for 24h. (TIF) [file pgen.1005563.s012.tif]

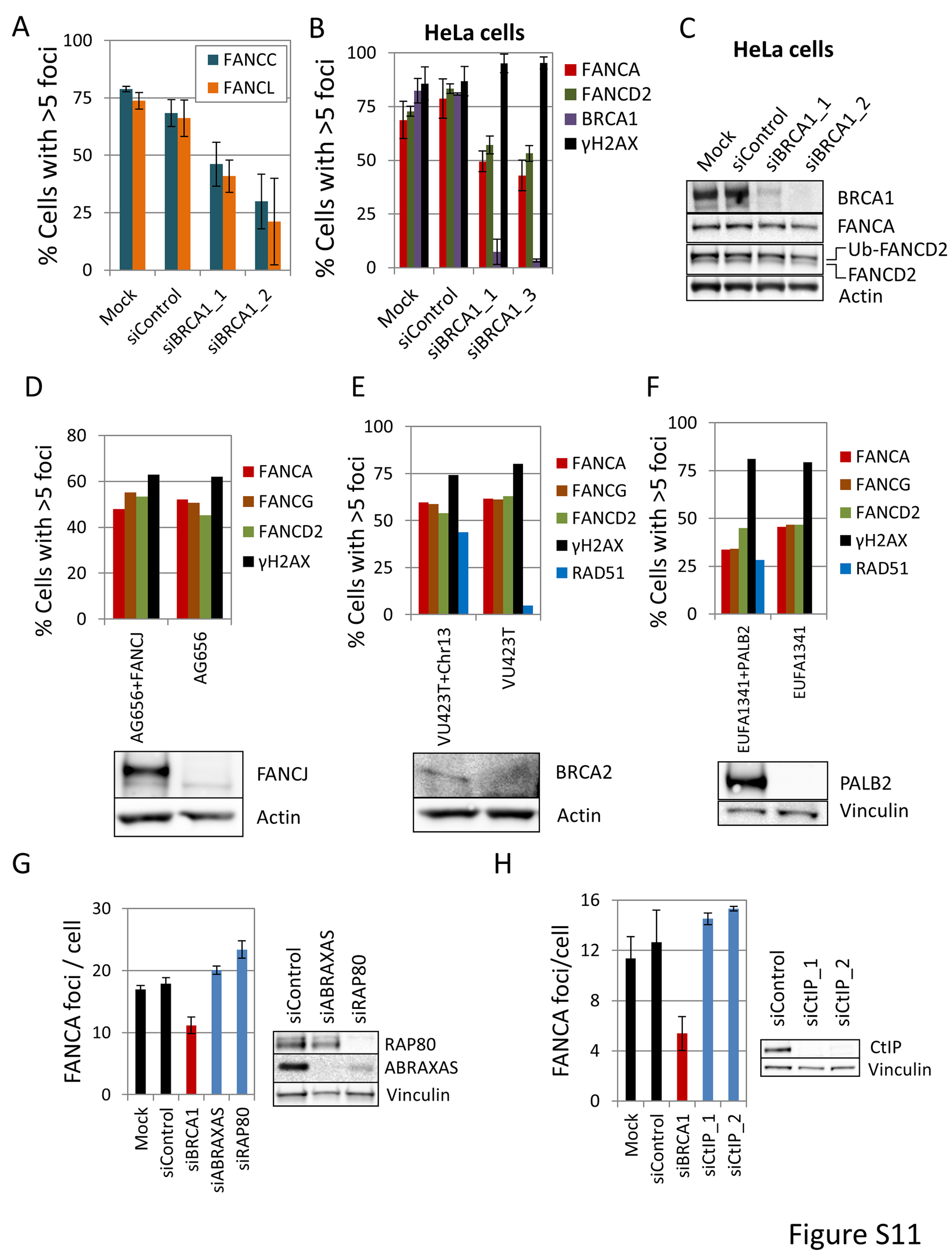

Supplement: S11 Fig — (A) U2OS cells were transfected with the indicated siRNAs, treated with MMC 60ng/ml for 24h., then fixed and immunostained with anti-FANCC and anti-FANCL antibodies. The percentage of cells with > 5 foci (n = 3, mean ± SD) is shown. (B) HeLa cells were transfected with the indicated siRNAs and treated with MMC 60ng/ml for 24h. The percentage of cells with > 5 foci (n = 3, mean ± SD) is shown. (C) Immunoblot analyses corresponding to the experiment described in panel A. (D) FANCJ-deficient AG656 and complemented fibroblasts treated with MMC 60ng/ml for 24 hours before fixation and staining. The percentage of cells with > 5 foci is shown in upper panel. Lower panel corresponds to western blotting confirming FANCJ status in these cell lines. (E) BRCA2-deficient VU423T and complemented fibroblasts were treated with MMC 60ng/ml for 24 hours before fixation and staining. Percentage of cells with > 5 foci is shown in upper panel. Lower panel corresponds to western blotting confirming BRCA2 status in these cell lines. (F) PALB2-deficient EUFA1341 and complemented fibroblasts were treated with MMC 60ng/ml for 24 hours before fixation and staining. Percentage of cells with > 5 foci is shown in upper panel. Lower panel corresponds to western blotting confirming PALB2 status in these cell lines. (G) and (H) U2OS cells were transfected with the indicated siRNAs and treated with MMC 60ng/ml for 24h. The number of foci/cell (n = 3, mean ± SD) is shown in left panels. Western blot confirming protein knock-down is shown in right panels. (TIF) [file pgen.1005563.s013.tif]

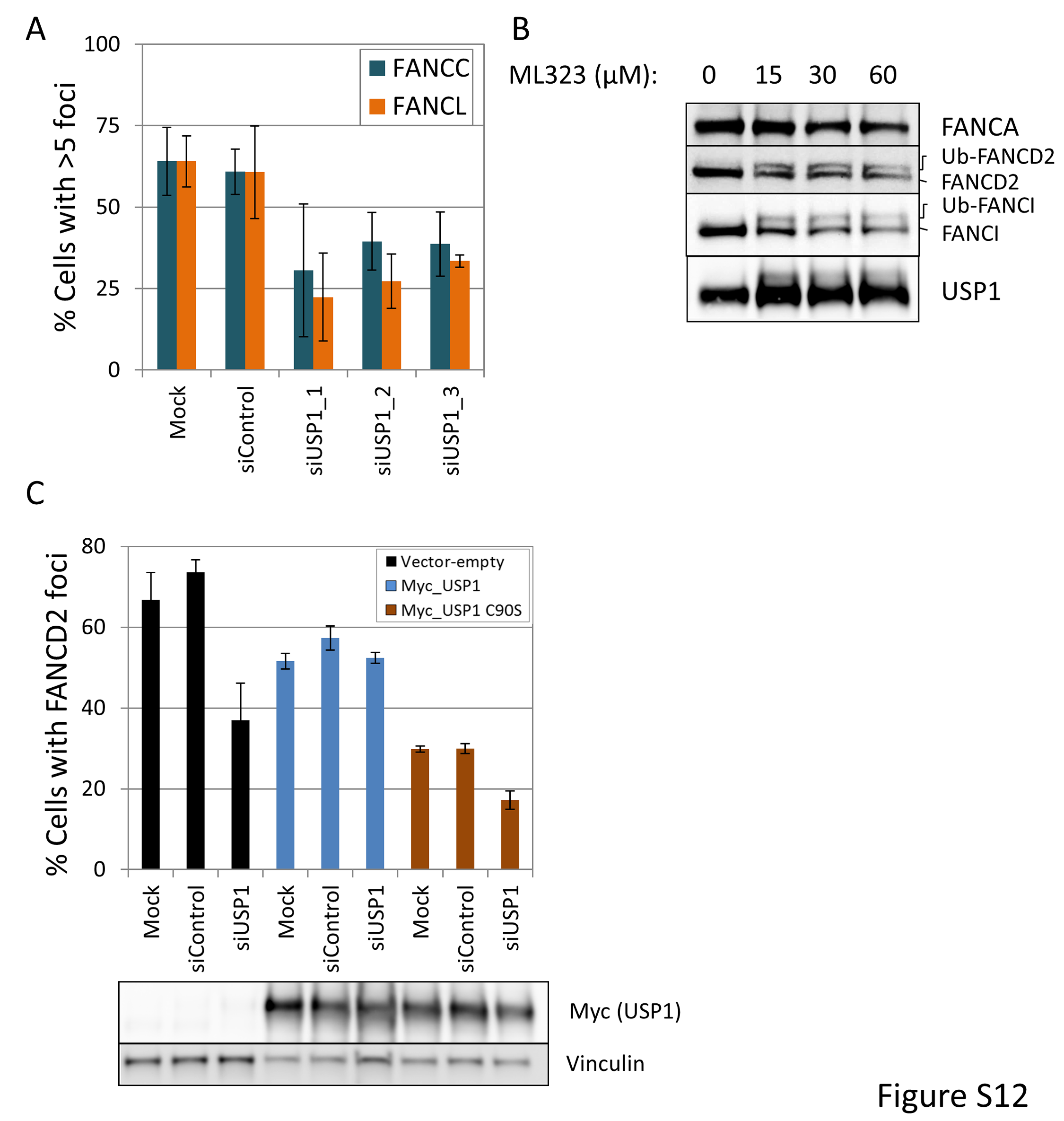

Supplement: S12 Fig — (A) U2OS cells were transfected with the indicated siRNAs, treated with IR 10 Gy, fixed 8 hours later, and immunostained with anti-FANCC and anti-FANCL antibodies. Percentage of cells with 5 or more foci is shown (n = 3, mean ± SD). (B) Immunoblot analyses corresponding to U2OS cells treated with the indicated doses of ML323 for 10 hours. (C) U2OS cells were transfected with the indicated combinations of siRNA and plasmid 48 hours before treatment (IR 10 Gy, fixed 8 hours later). The percentage of cells containing > 5 FANCD2 foci (n = 3, mean ± SD) is shown. In samples transfected with myc-tagged USP1 wild-type or myc-tagged USP1 C90S, only USP1-expressing cells (myc-positive) were included in the analysis. Immunoblot analyses corresponding to experiment shown in Fig 7C and 7D and S11C Fig. (TIF) [file pgen.1005563.s014.tif]

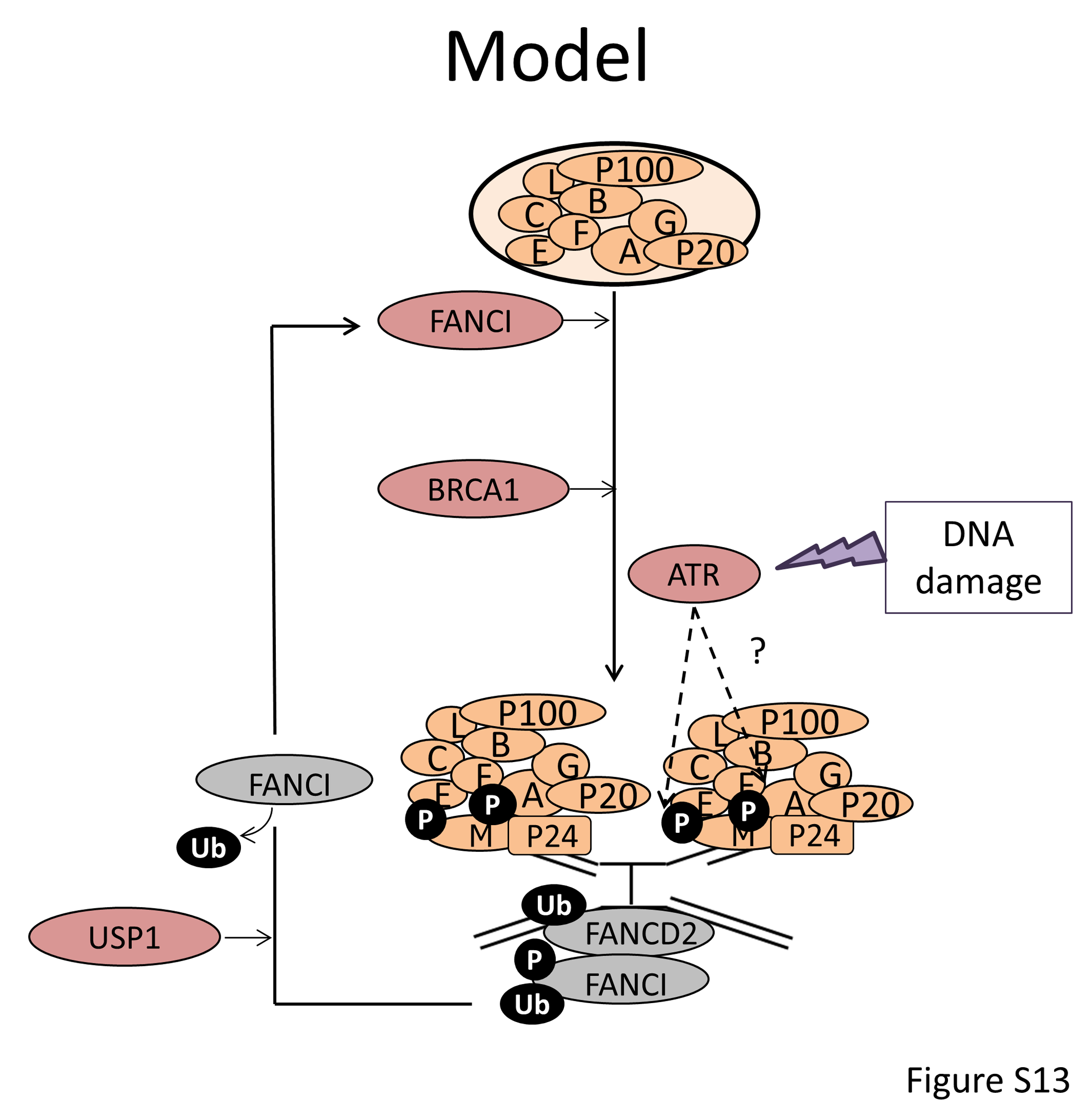

Supplement: S13 Fig — FANCI, BRCA1, USP1 and ATR positively regulate FA core complex foci formation. Non-ubiquitinated/non-phosphorylated FANCI is able to perform this function, independently of FANCD2. USP1 promotes FA core complex foci by deubiquitinating FANCI. ATR kinase activity is required. Candidate substrates include FANCM, FANCA or other members of the FA core complex. At the site of DNA damage, the FA core complex may help promote recruitment of ubiquitinated FANCD2-FANCI. (TIF) [file pgen.1005563.s015.tif]
